# Supplementary figures and images for: Mycobacterium tuberculosis impairs protective cytokine production via transcription factor MafB manipulation
Source: PLoS Pathog. 2025 Sep 4;21(9):e1013476. doi: 10.1371/journal.ppat.1013476 (PMC12419584; doi:10.1371/journal.ppat.1013476)

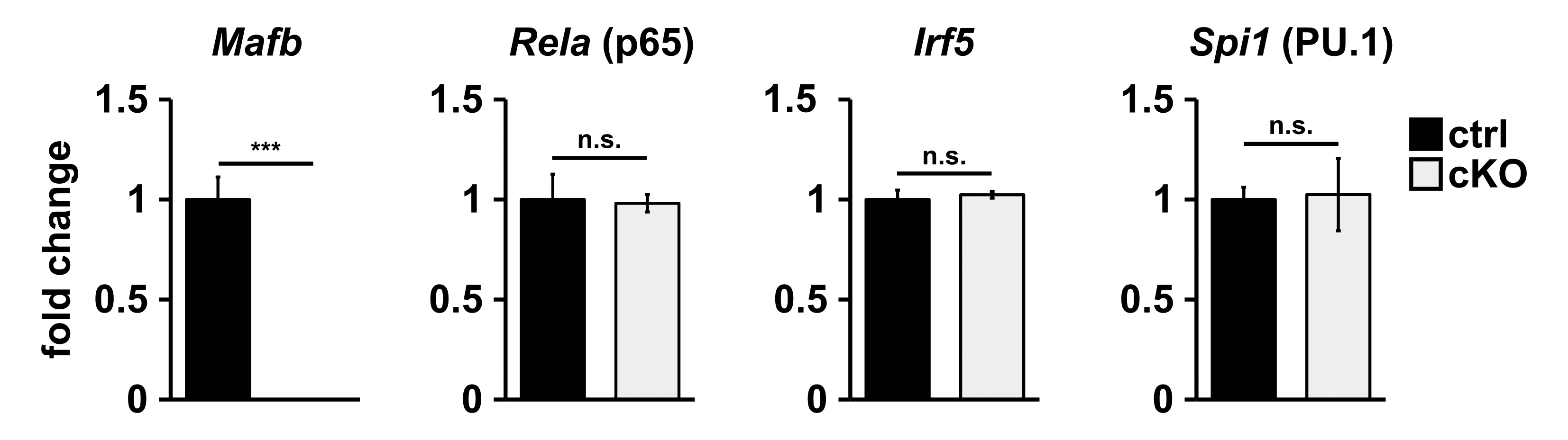

Supplement: S1 Fig — Mice were intraperitoneally injected with 4% thioglycolate, and macrophages were isolated from the peritoneal cavity three days later. Cells were harvested and analyzed using quantitative RT–PCR. Relative gene expression is represented as a fold increase above background level of samples prepared from control cells. Data are representative of two independent experiments. ***P < 0.001; n.s., not significant; mean ± SD. (TIF) [file ppat.1013476.s001.tif]

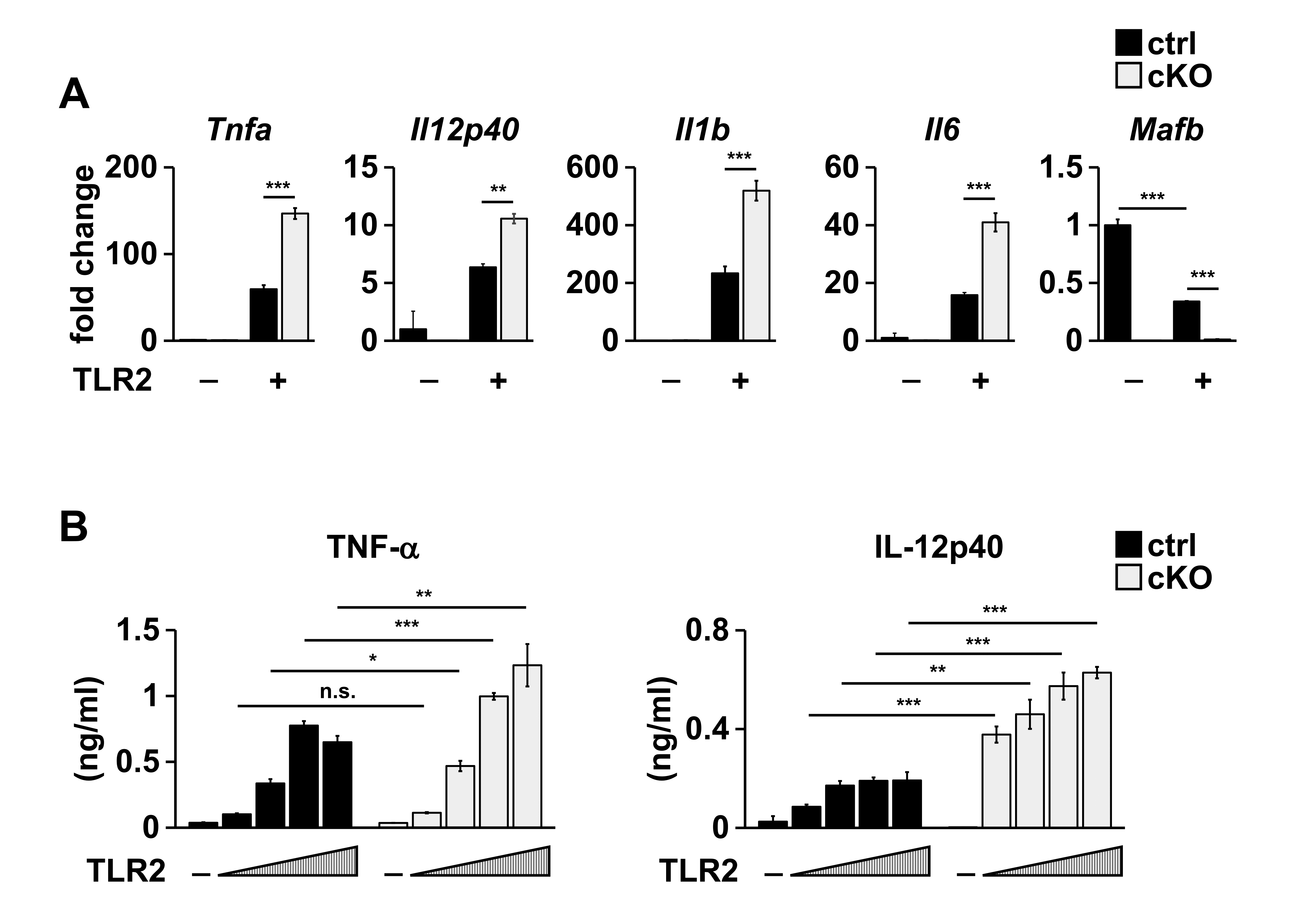

Supplement: S2 Fig — (A) Mice were intraperitoneally injected with 4% thioglycolate, and macrophages were isolated from the peritoneal cavity three days later. Following a 6-h stimulation with 100 ng/mL Pam3CSK4, cells were harvested and analyzed using quantitative RT–PCR. Relative gene expression is represented as a fold increase above background level of samples prepared from unstimulated cells. Data are representative of three independent experiments. **P < 0.01; ***P < 0.001; mean ± SD. (B) Mice were intraperitoneally injected with 4% thioglycolate. Three days later, macrophages were isolated from the peritoneal cavity. Following a 6-h stimulation with 0, 100, 200, 400, or 500 ng/mL Pam3CSK4, cytokine production was measured using ELISA. Data are representative of three independent experiments. *P < 0.05; **P < 0.01; ***P < 0.001; n.s., not significant; mean ± SD. (TIF) [file ppat.1013476.s002.tif]

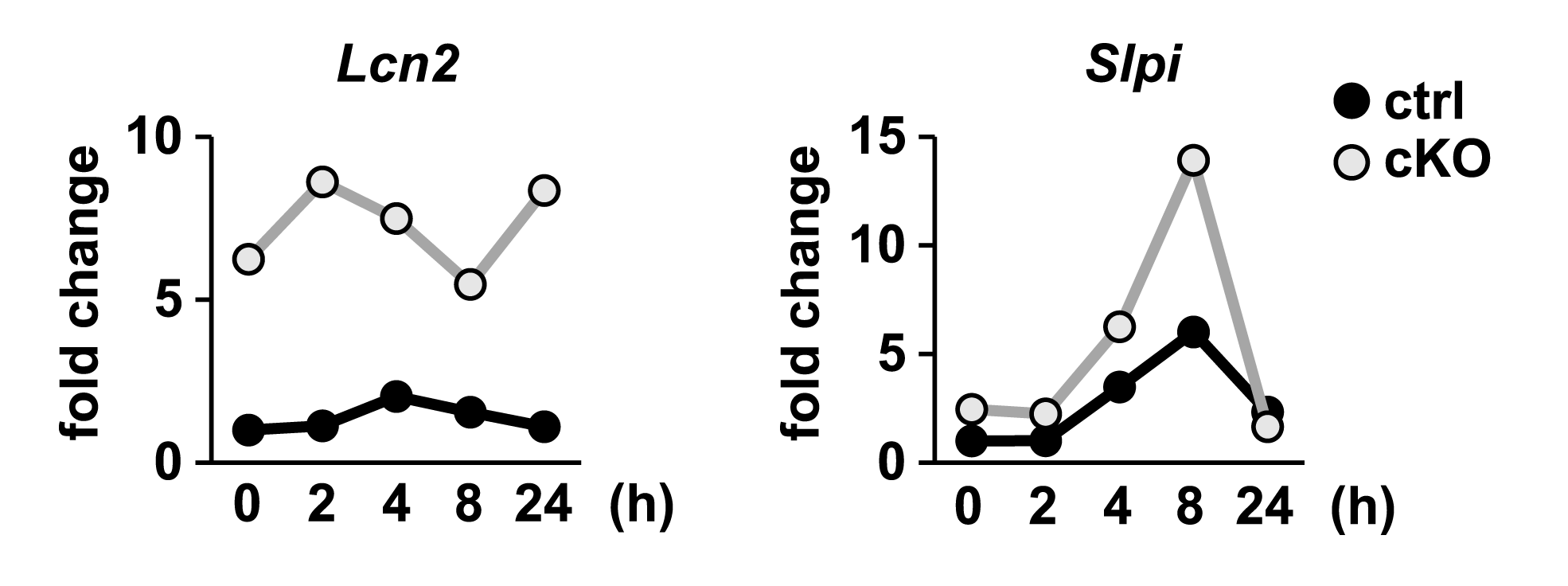

Supplement: S3 Fig — Mice were intraperitoneally injected with 4% thioglycolate, and macrophages were isolated from the peritoneal cavity three days later. At the indicated time points after infection with M. tuberculosis (MOI of 5), cells were harvested and analyzed using quantitative RT–PCR. Relative gene expression is represented as a fold increase above background level of samples prepared from uninfected control cells. Data are representative of three independent experiments. (TIF) [file ppat.1013476.s003.tif]

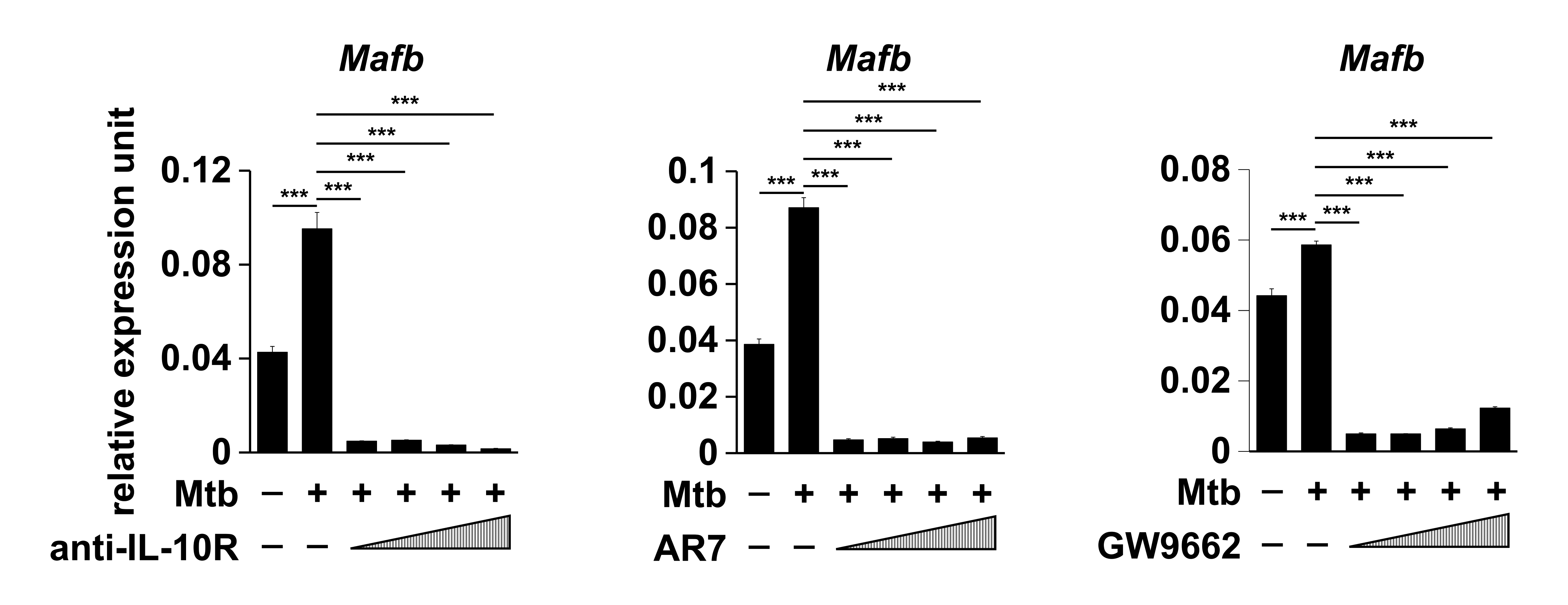

Supplement: S4 Fig — Mice were intraperitoneally injected with 4% thioglycolate, and macrophages were isolated from the peritoneal cavity three days later. Cells were infected with M. tuberculosis H37Rv (MOI of 5) after priming with anti-mouse IL-10R (0, 10, 20, 50, or 100 μg/mL), AR7 (RAR inhibitor; 0, 10, 20, 50, or 100 μM), or GW9662 (PPAR-γ inhibitor; 0, 10, 20, 50, or 100 μM). Six hours following infection, cells were harvested and analyzed using quantitative RT–PCR. Mafb mRNA level is normalized by the corresponding Gapdh level. Data are representative of two independent experiments. ***P < 0.001; mean ± SD. (TIF) [file ppat.1013476.s004.tif]

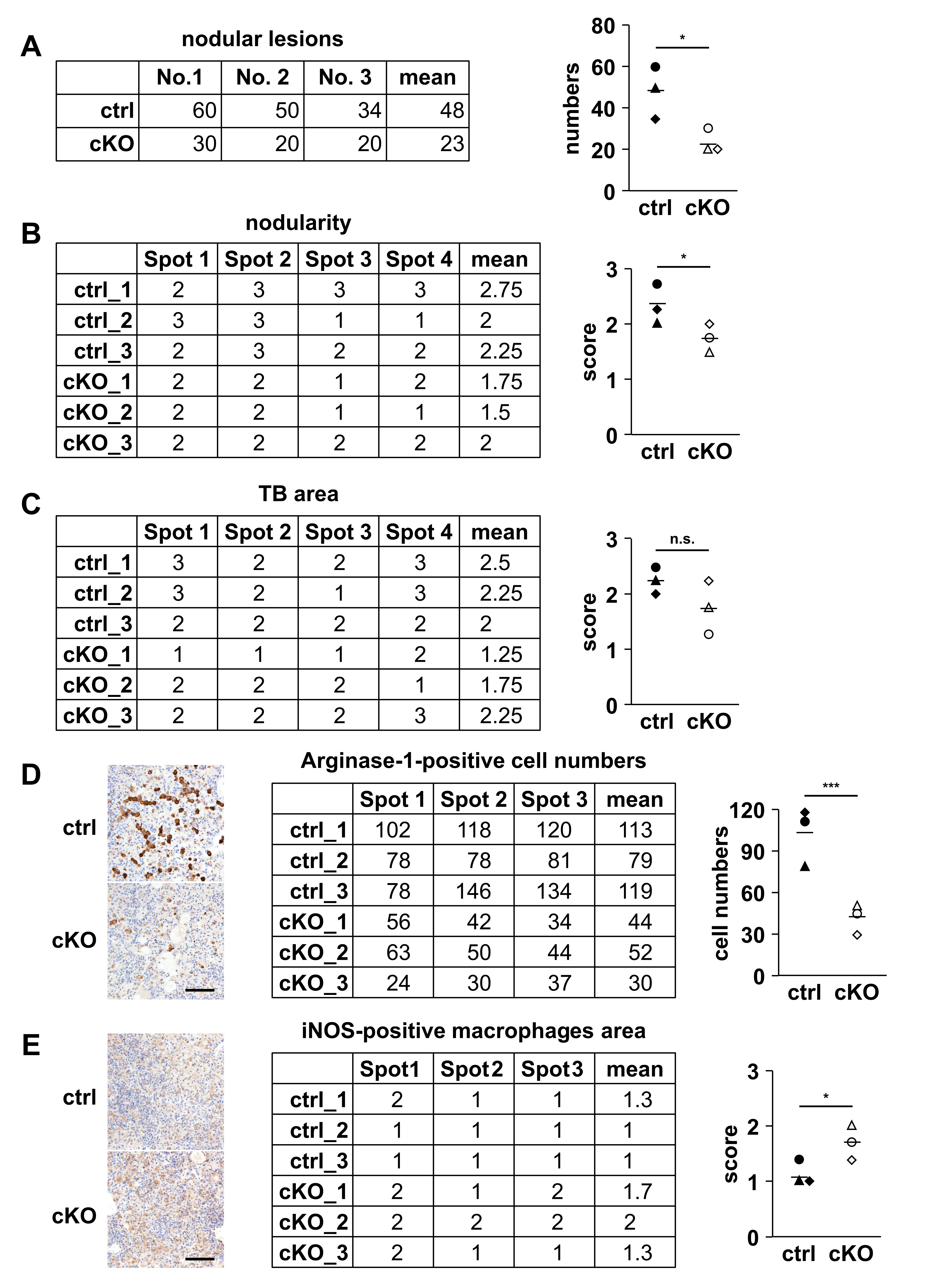

Supplement: S5 Fig — Mafbf/f (n = 3) and Mafbf/f::LysM-Cre (n = 3) mice were intratracheally infected with M. tuberculosis H37Rv (5 × 10^3^ CFU). Three weeks after infection, the lungs were fixed in paraffin, and lung tissue sections were used for several staining procedures. (A) The number of nodular lesions was determined using hematoxylin-eosin-stained lung sections. *P < 0.05. (B) The degree of nodularity was scored using Iba1 immunohistochemical staining panels. 3: almost nodal, 2: nodal predominance, 1: diffuse predominance. *P < 0.05. (C) The degree of TB area was scored using Ziehl-Neelsen staining panels. 3: positive in areas > 50%, 2: positive in the 25%–50% range, 1: positive in areas < 25%. n.s., not significant. (D) The number of Arginase-1-positive macrophages was determined using Arginase-1 immunohistochemical staining panels (Scale bar represents 100 μm). ***P < 0.001. (E) The degree of iNOS-positive macrophages area was scored using iNOS immunohistochemical staining panels (Scale bar represents 100 μm). 2: positive in areas > 50%, 1: positive in areas < 50%. *P < 0.05. (TIF) [file ppat.1013476.s005.tif]
