# Supplementary material for: Mycobacterium tuberculosis impairs protective cytokine production via transcription factor MafB manipulation
Source: PLoS Pathog. 2025 Sep 4;21(9):e1013476. doi: 10.1371/journal.ppat.1013476 (PMC12419584; doi:10.1371/journal.ppat.1013476)
Supplement: S2 Data — This file includes raw image data of Figs 2A, 2B, 2C, 2E, 2F, 3A, 3C, 3E, 4B, 4E, 5E, 5F, S5A, S5B, S5C, S5D, and S5E. (PDF) [file ppat.1013476.s007.pdf]

Fig 2A

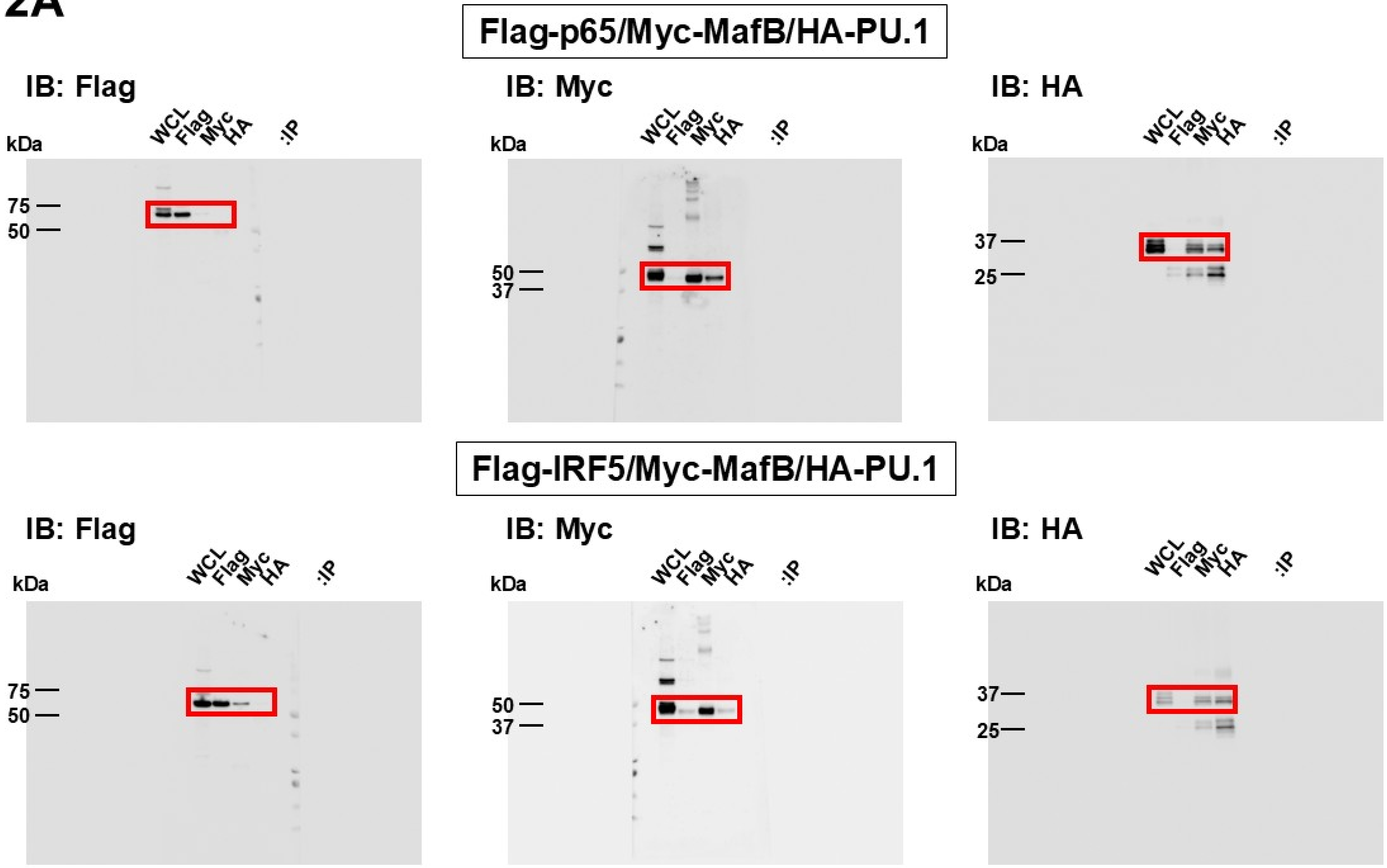
Flag-p65/Myc-MafB/HA-PU.1
IB: Flag
IB: Myc
IB: HA
WCL
Flag
Myc
HA
:IP
kDa
75
50
50
37
37
25
Flag-IRF5/Myc-MafB/HA-PU.1
IB: Flag
IB: Myc
IB: HA
WCL
Flag
Myc
HA
:IP
kDa
75
50
50
37
37
25

Fig 2B

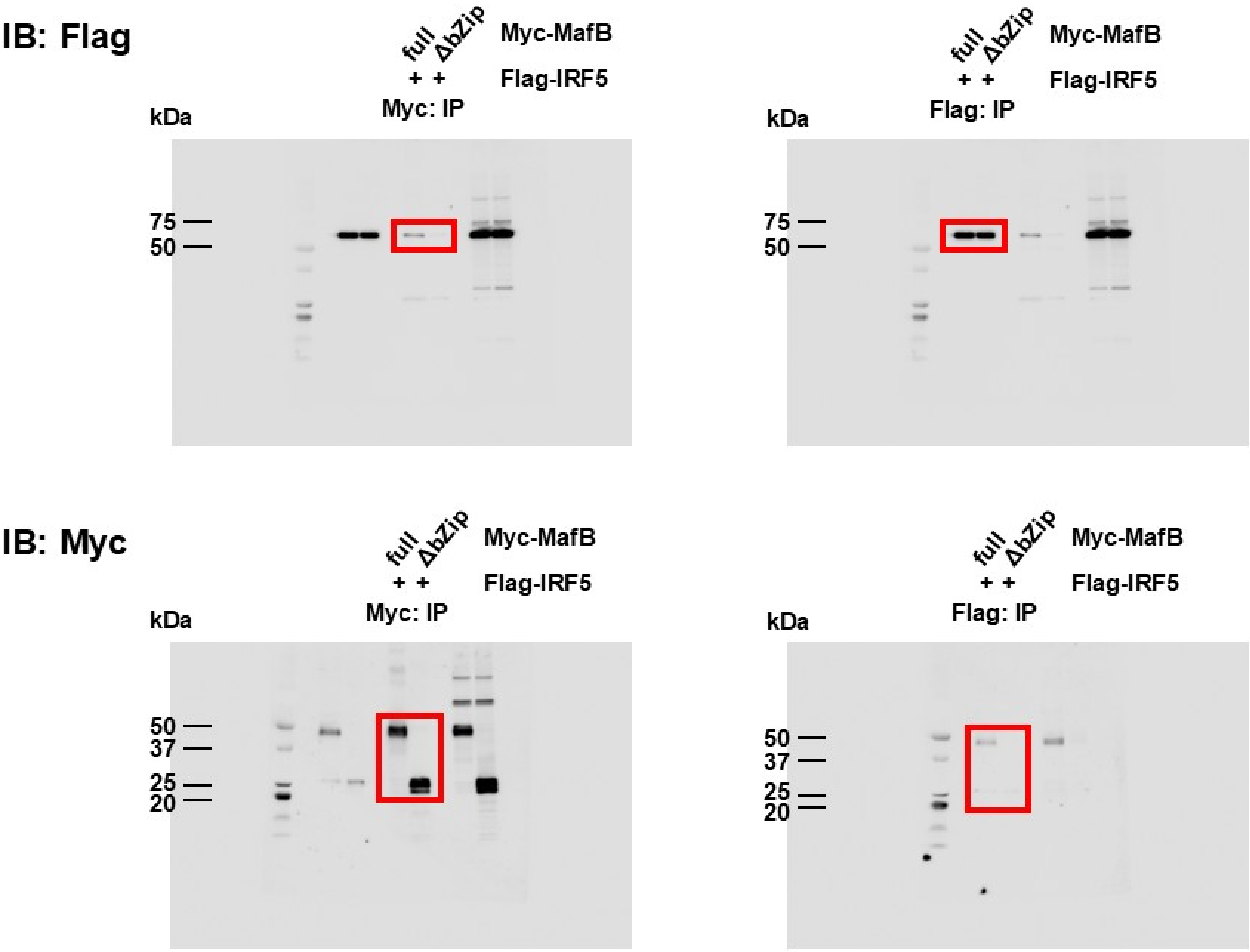
IB: Flag
full
ΔbZip
Myc-MafB
+ +
Flag-IRF5
Myc: IP
kDa
75
50
full
ΔbZip
Myc-MafB
+ +
Flag-IRF5
Flag: IP
kDa
75
50
IB: Myc
full
ΔbZip
Myc-MafB
+ +
Flag-IRF5
Myc: IP
kDa
50
37
25
20
full
ΔbZip
Myc-MafB
+ +
Flag-IRF5
Flag: IP
kDa
50
37
25
20

Fig 2C

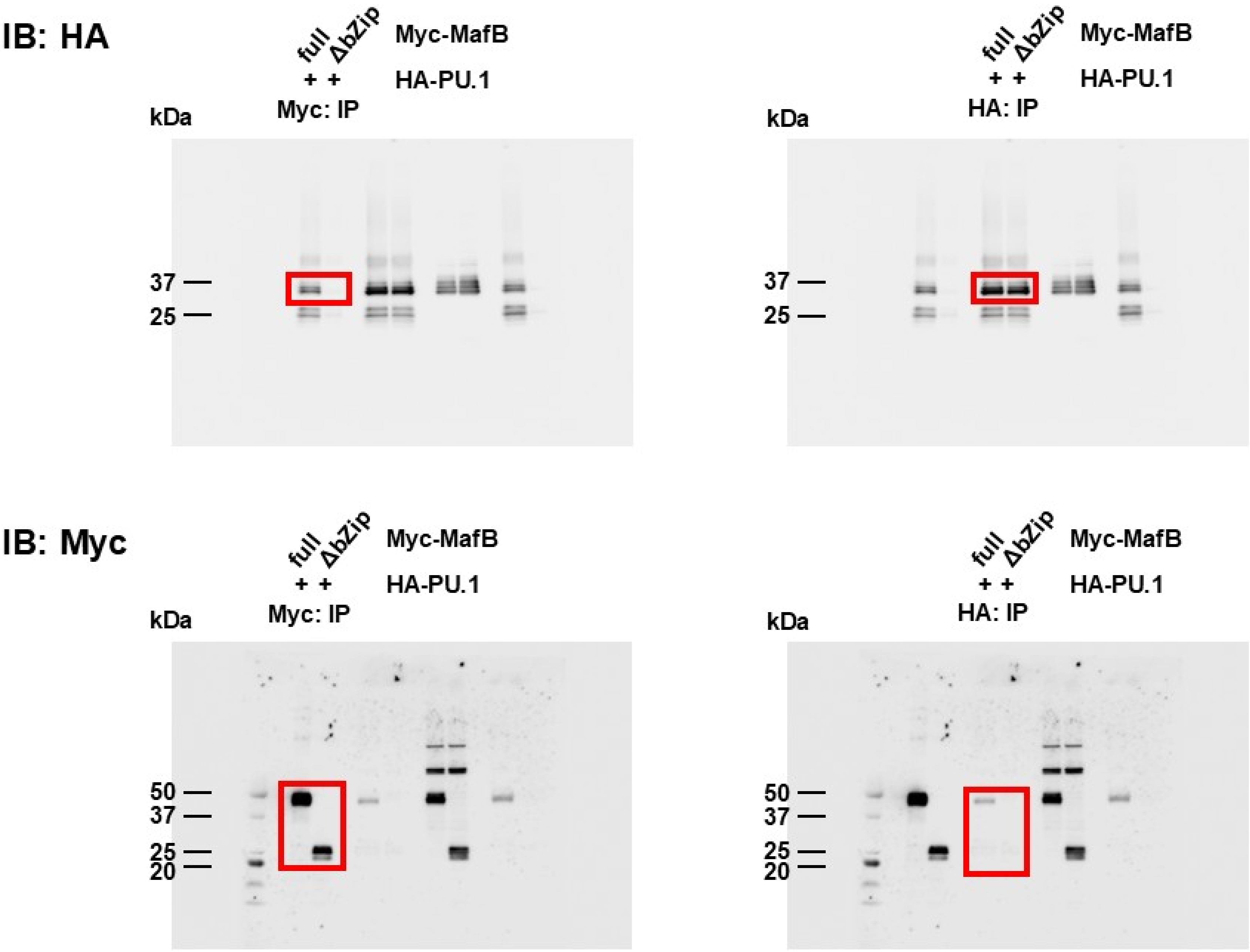
IB: HA
full
ΔbZip
Myc-MafB
+ +
HA-PU.1
Myc: IP
kDa
37
25
full
ΔbZip
Myc-MafB
+ +
HA-PU.1
HA: IP
kDa
37
25
IB: Myc
full
ΔbZip
Myc-MafB
+ +
HA-PU.1
Myc: IP
kDa
50
37
25
20
full
ΔbZip
Myc-MafB
+ +
HA-PU.1
HA: IP
kDa
50
37
25
20

Fig 2E

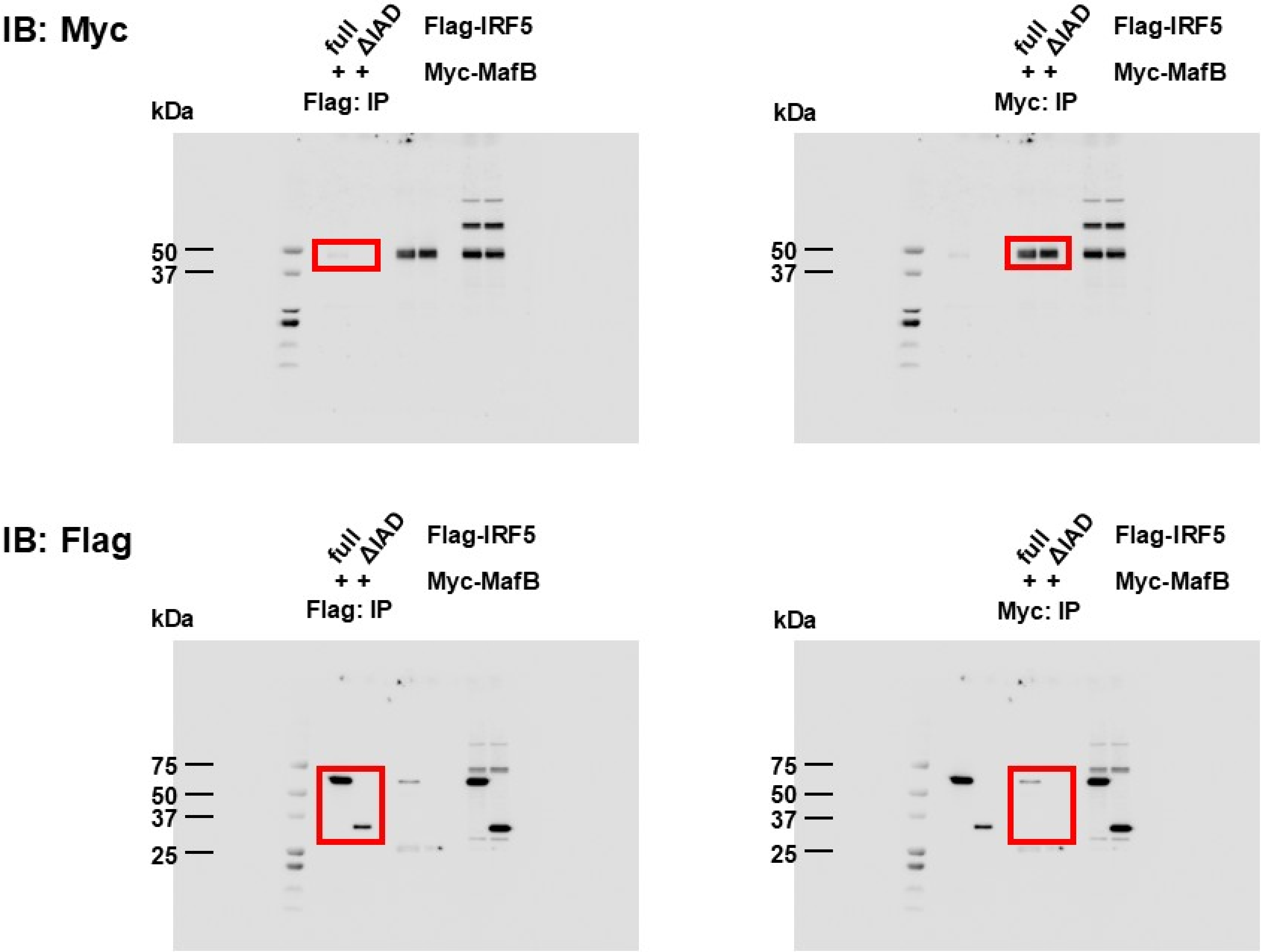
IB: Myc
full
ΔIAD
Flag-IRF5
+ +
Myc-MafB
Flag: IP
kDa
50
37
full
ΔIAD
Flag-IRF5
+ +
Myc-MafB
Myc: IP
kDa
50
37
IB: Flag
full
ΔIAD
Flag-IRF5
+ +
Myc-MafB
Flag: IP
kDa
75
50
37
25
full
ΔIAD
Flag-IRF5
+ +
Myc-MafB
Myc: IP
kDa
75
50
37
25

Fig 2F

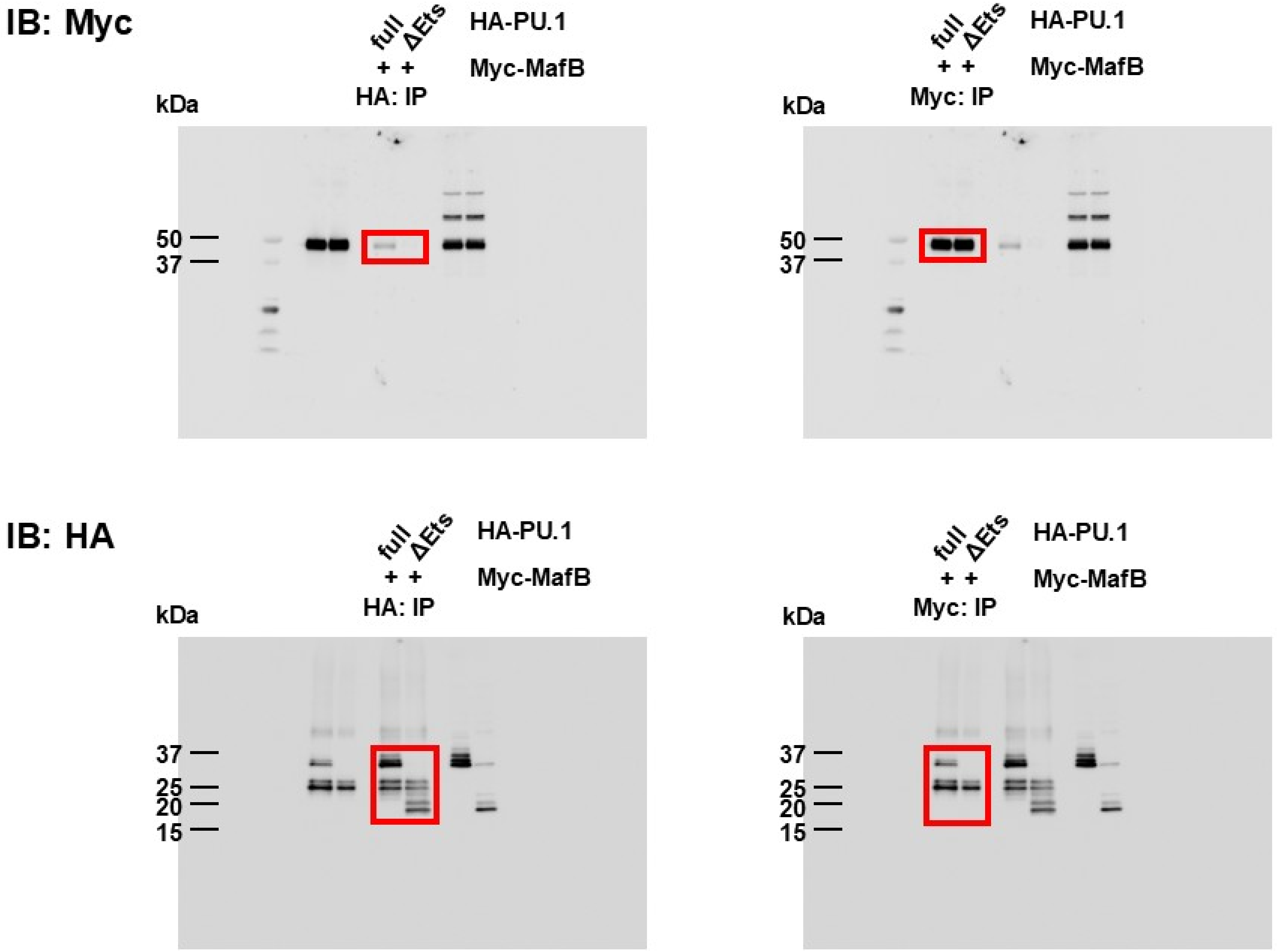
IB: Myc
full
ΔEts
HA-PU.1
+ +
Myc-MafB
HA: IP
kDa
50
37
full
ΔEts
HA-PU.1
+ +
Myc-MafB
Myc: IP
kDa
50
37
IB: HA
full
ΔEts
HA-PU.1
+ +
Myc-MafB
HA: IP
kDa
37
25
20
15
full
ΔEts
HA-PU.1
+ +
Myc-MafB
Myc: IP
kDa
37
25
20
15

Fig 3A

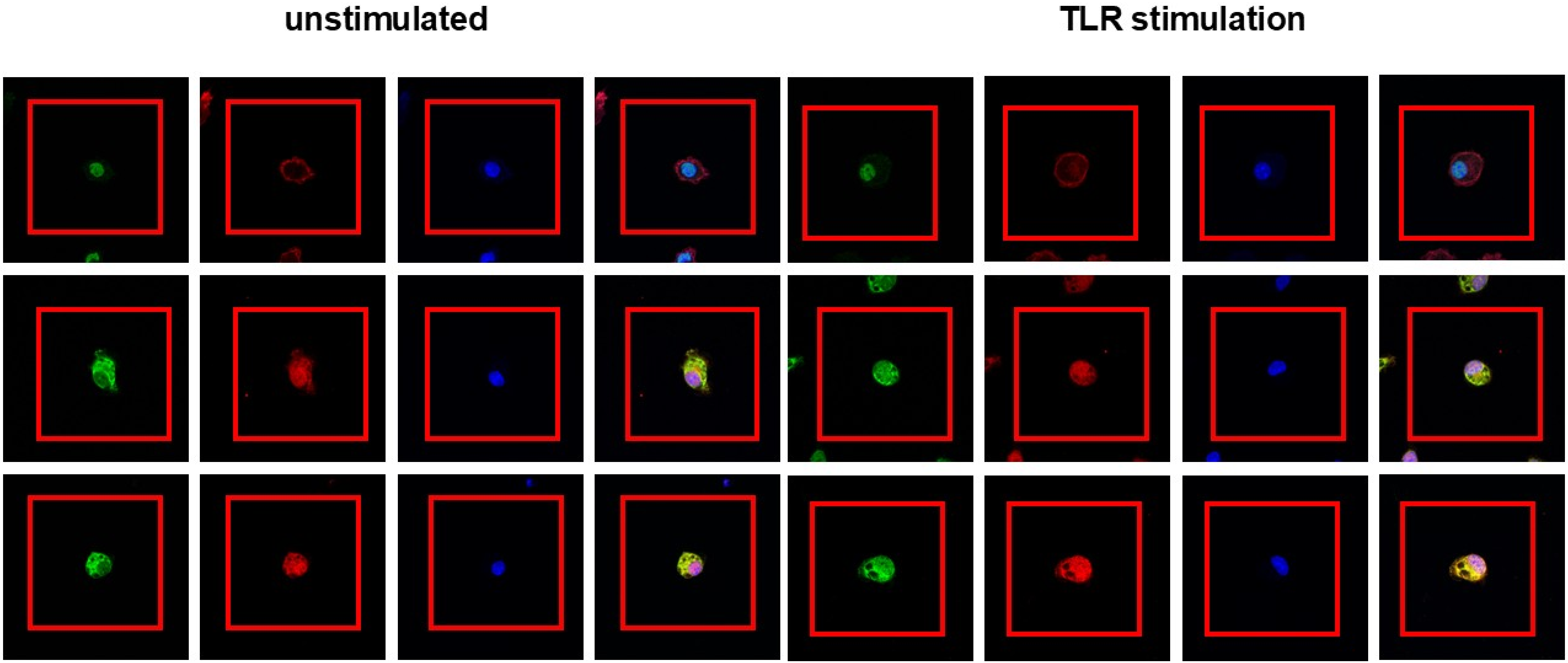

Fig 3C

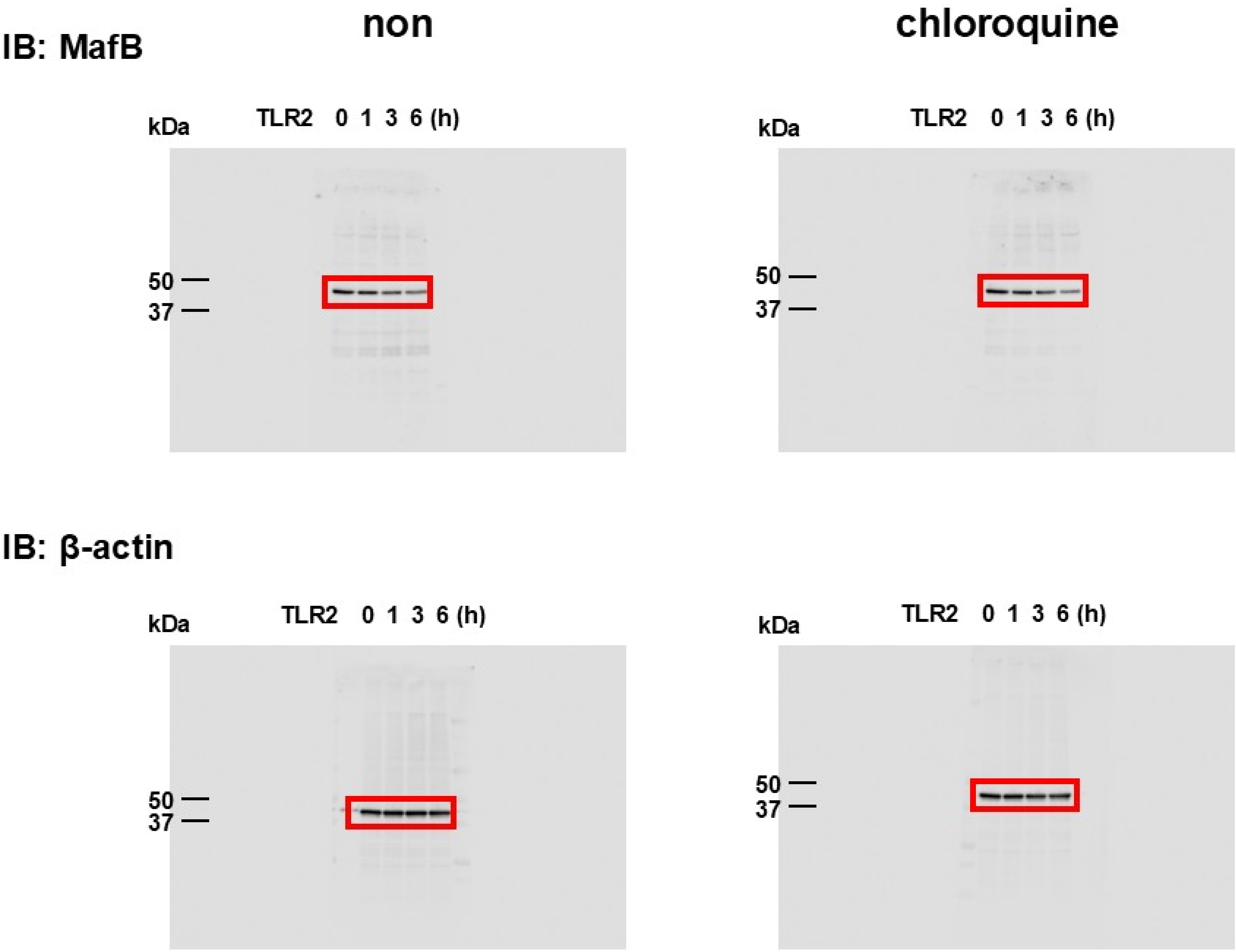
non
chloroquine
IB: MafB
kDa
TLR2 0 1 3 6 (h)
50
37
kDa
TLR2 0 1 3 6 (h)
50
37
IB: β-actin
kDa
TLR2 0 1 3 6 (h)
50
37
kDa
TLR2 0 1 3 6 (h)
50
37

Fig 3C

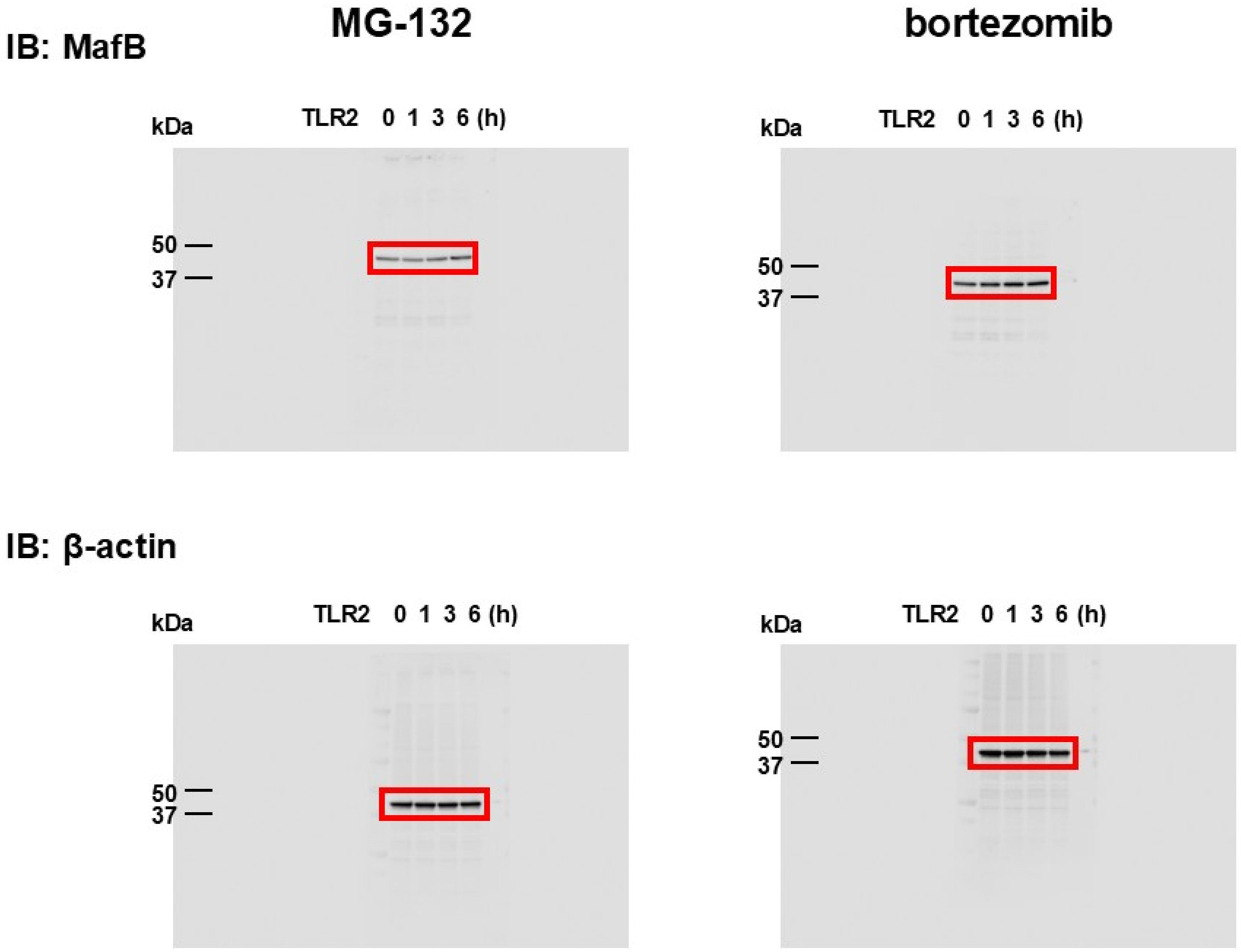
MG-132
bortezomib
IB: MafB
kDa
TLR2 0 1 3 6 (h)
50
37
kDa
TLR2 0 1 3 6 (h)
50
37
IB: β-actin
kDa
TLR2 0 1 3 6 (h)
50
37
kDa
TLR2 0 1 3 6 (h)
50
37

Fig 3E

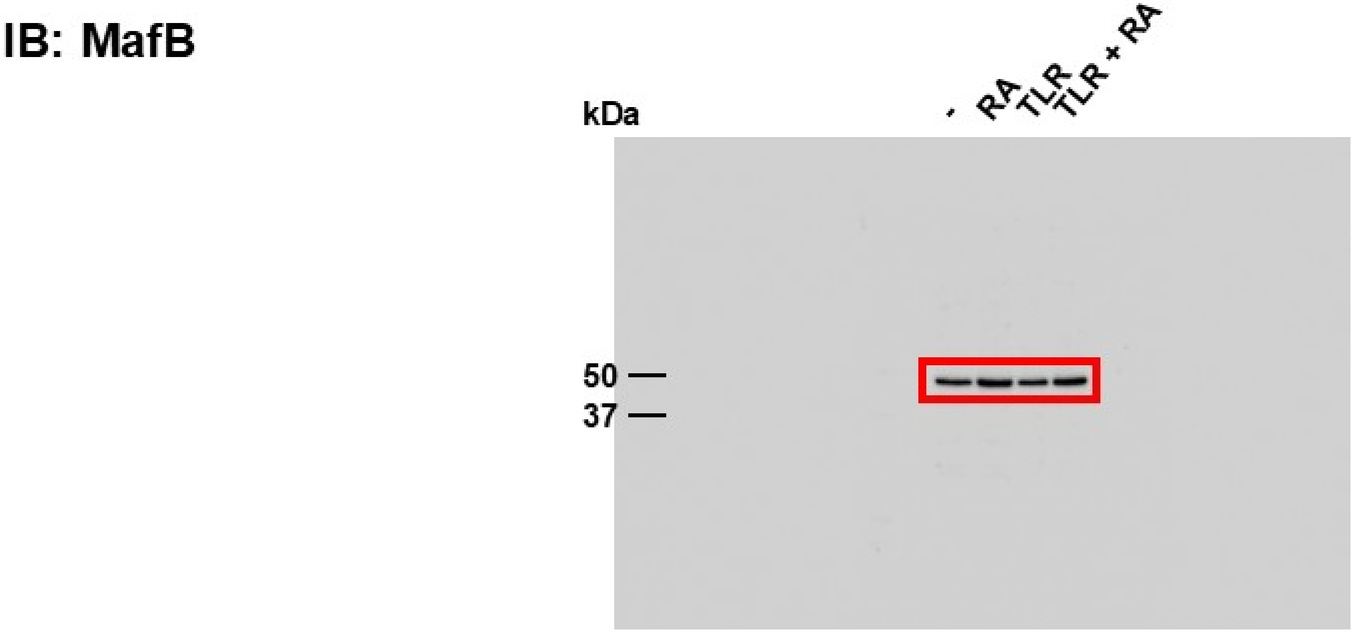

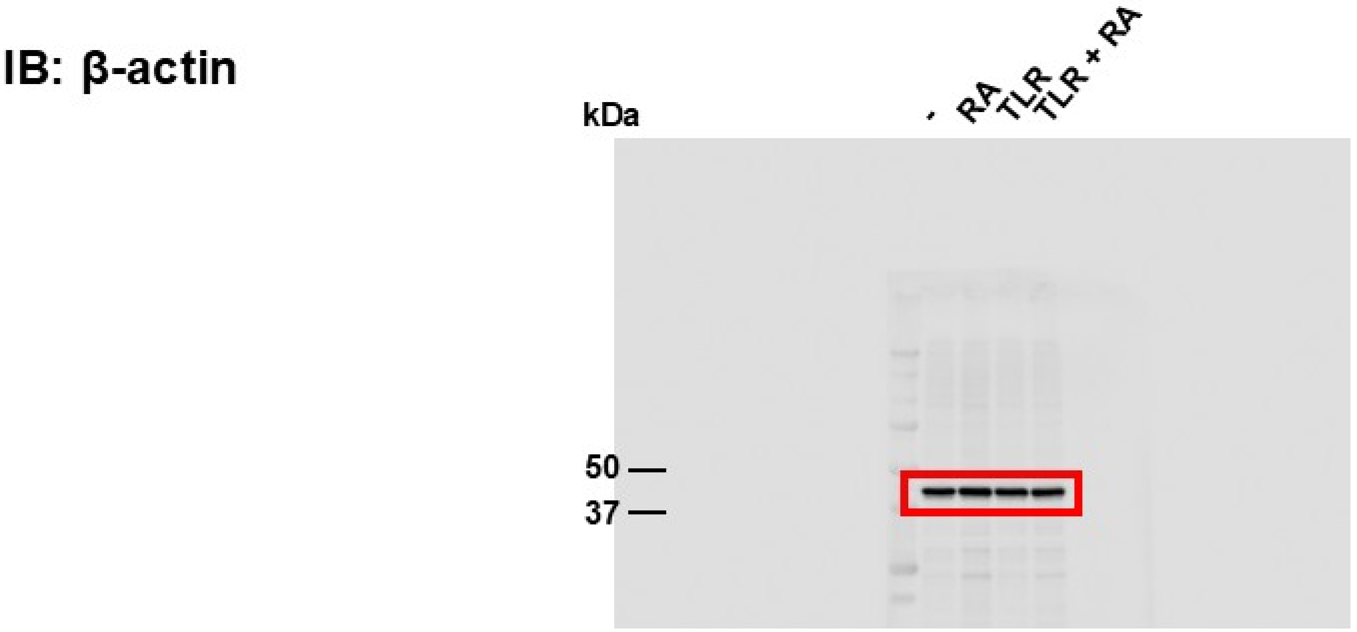

Fig 4B

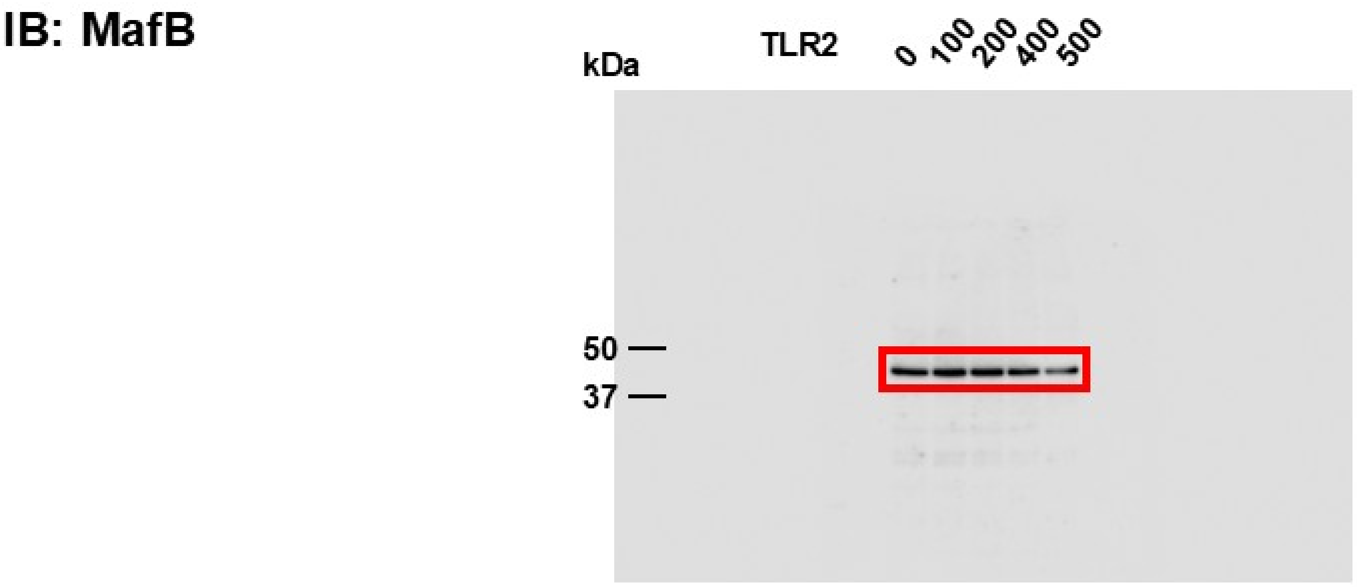
IB: MafB
kDa
TLR2
0
100
200
400
500
50
37

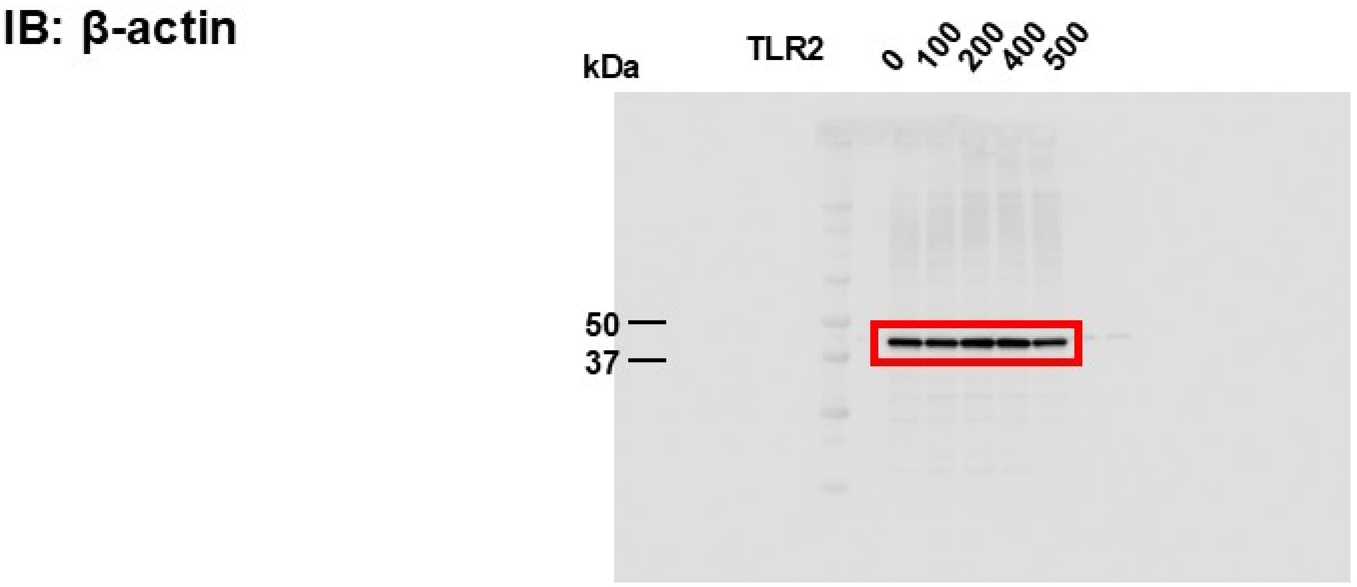
IB: β-actin
kDa
TLR2
0
100
200
400
500
50
37

Fig 4E

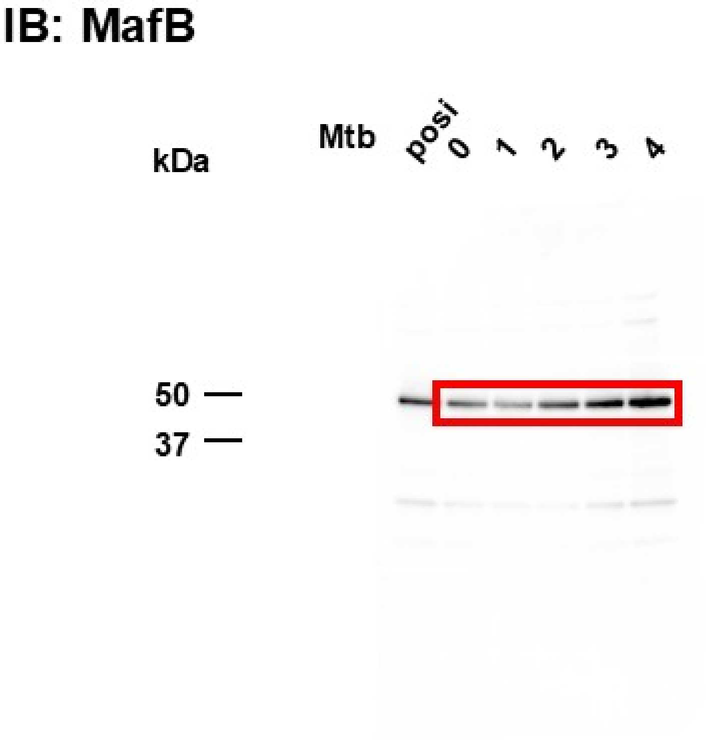
IB: MafB
kDa
Mtb
posi
0
1
2
3
4
50
37

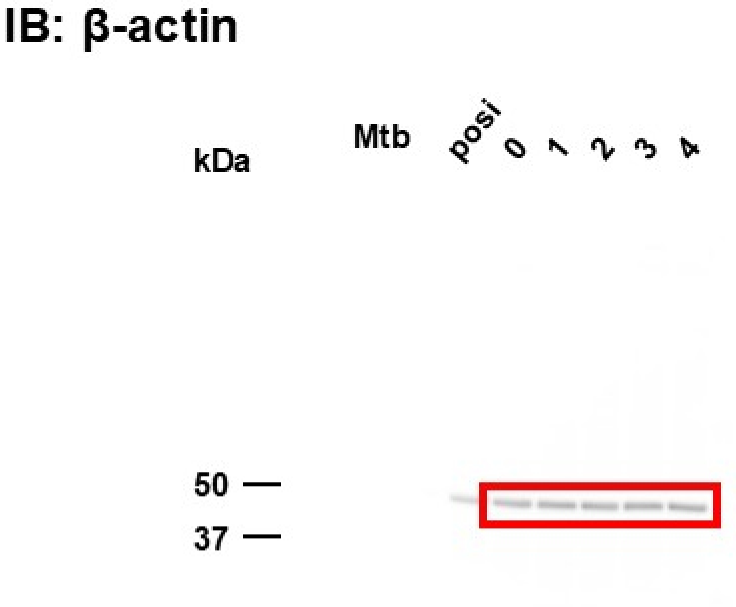
IB: β-actin
kDa
Mtb
posi
0
1
2
3
4
50
37

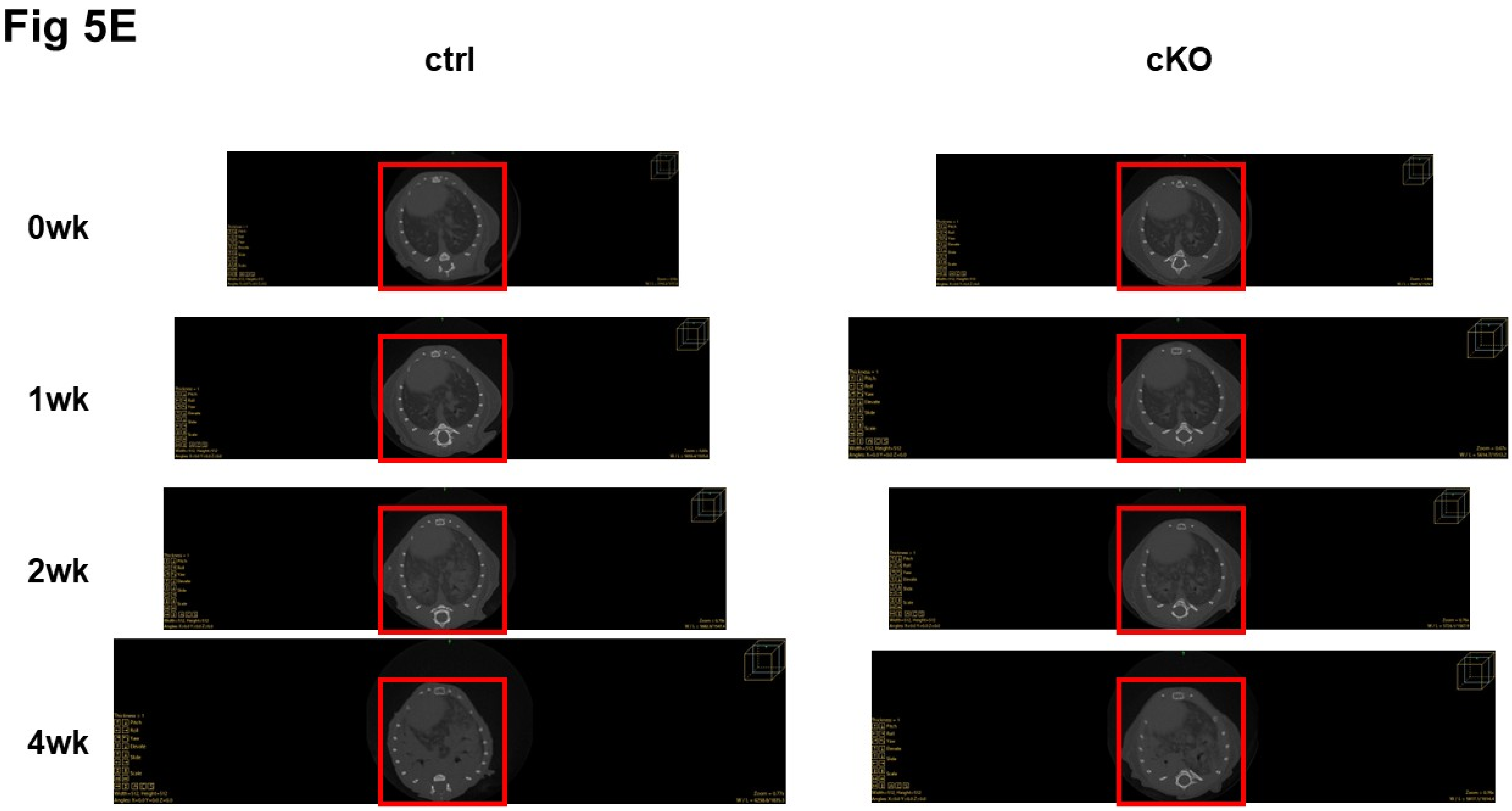
Fig 5E
ctrl
cKO
0wk
1wk
2wk
4wk

Fig 5F

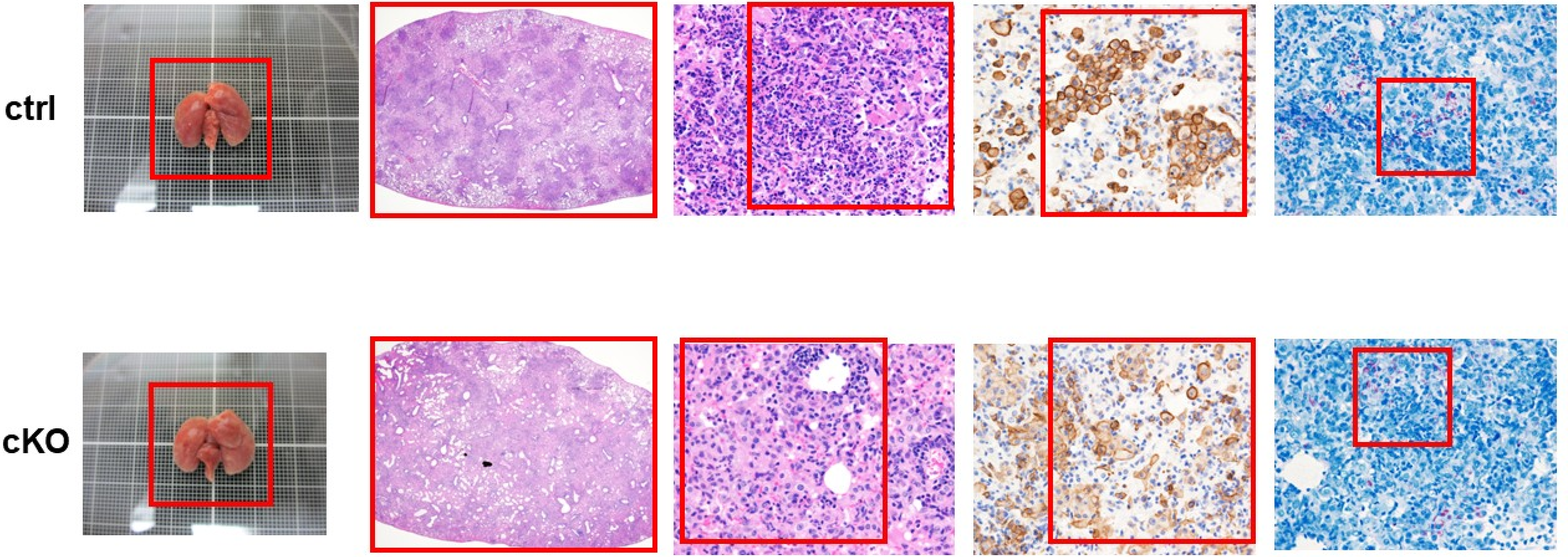

S5A Fig

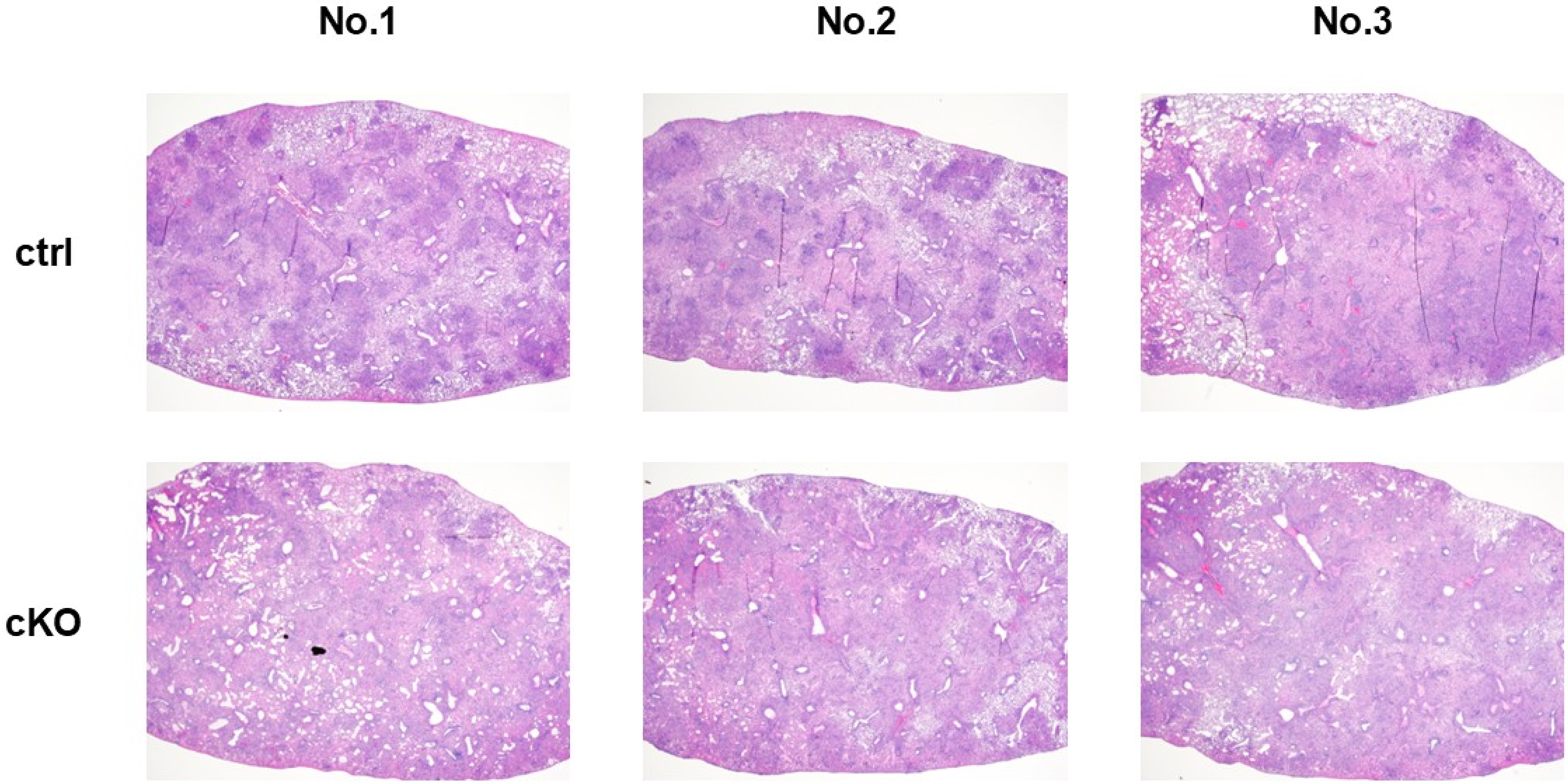

S5B Fig

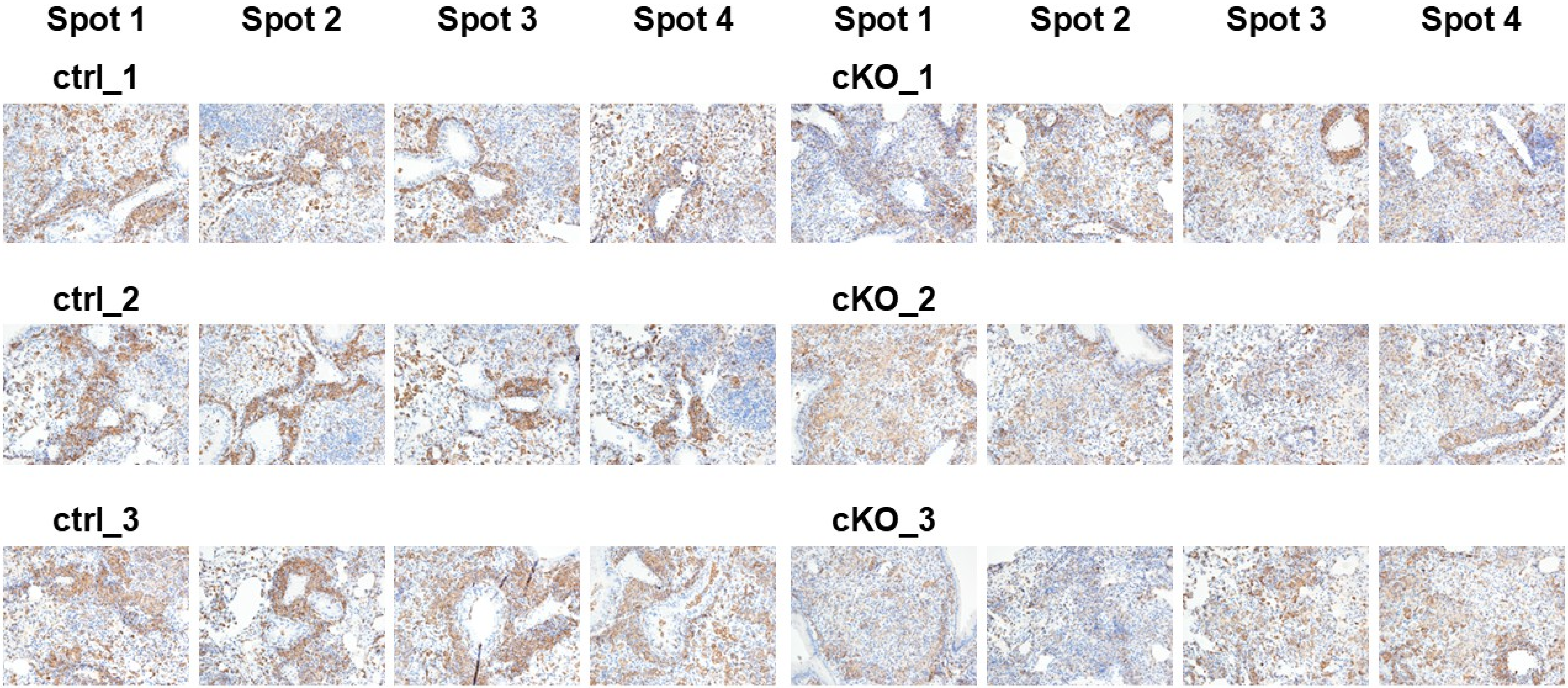
Spot 1
Spot 2
Spot 3
Spot 4
Spot 1
Spot 2
Spot 3
Spot 4
ctrl_1
cKO_1
ctrl_2
cKO_2
ctrl_3
cKO_3

S5C Fig

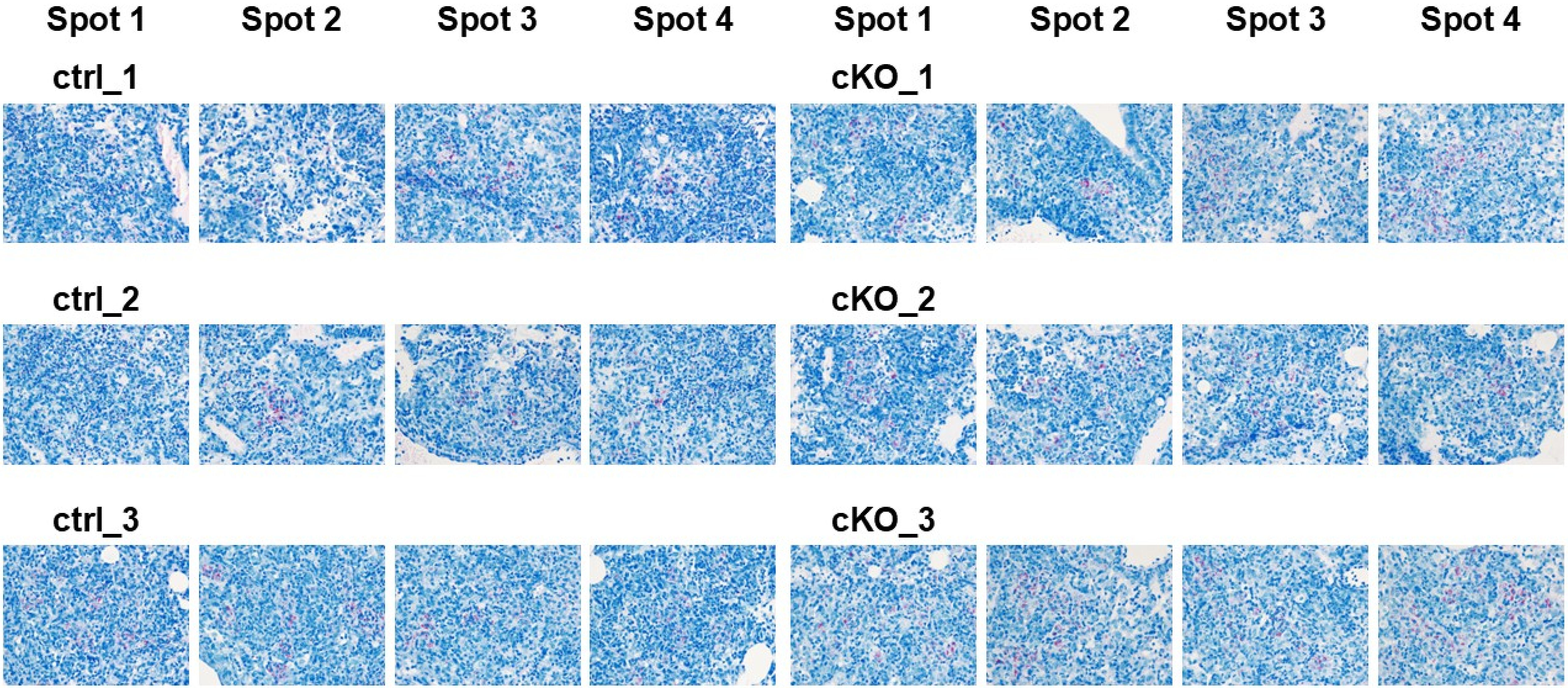
Spot 1
Spot 2
Spot 3
Spot 4
Spot 1
Spot 2
Spot 3
Spot 4
ctrl_1
cKO_1
ctrl_2
cKO_2
ctrl_3
cKO_3

S5D Fig

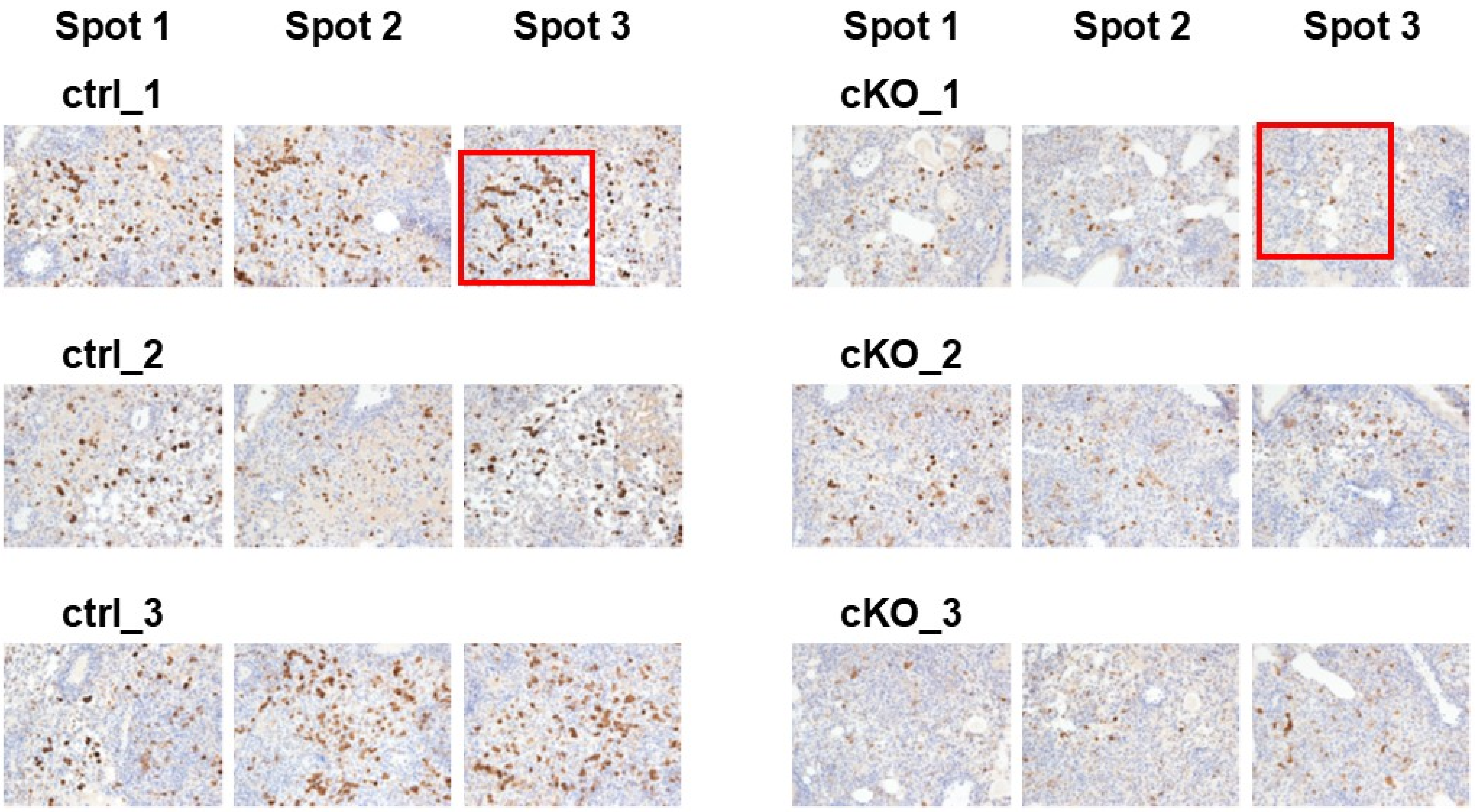
Spot 1
Spot 2
Spot 3
Spot 1
Spot 2
Spot 3
ctrl_1
cKO_1
ctrl_2
cKO_2
ctrl_3
cKO_3

S5E Fig

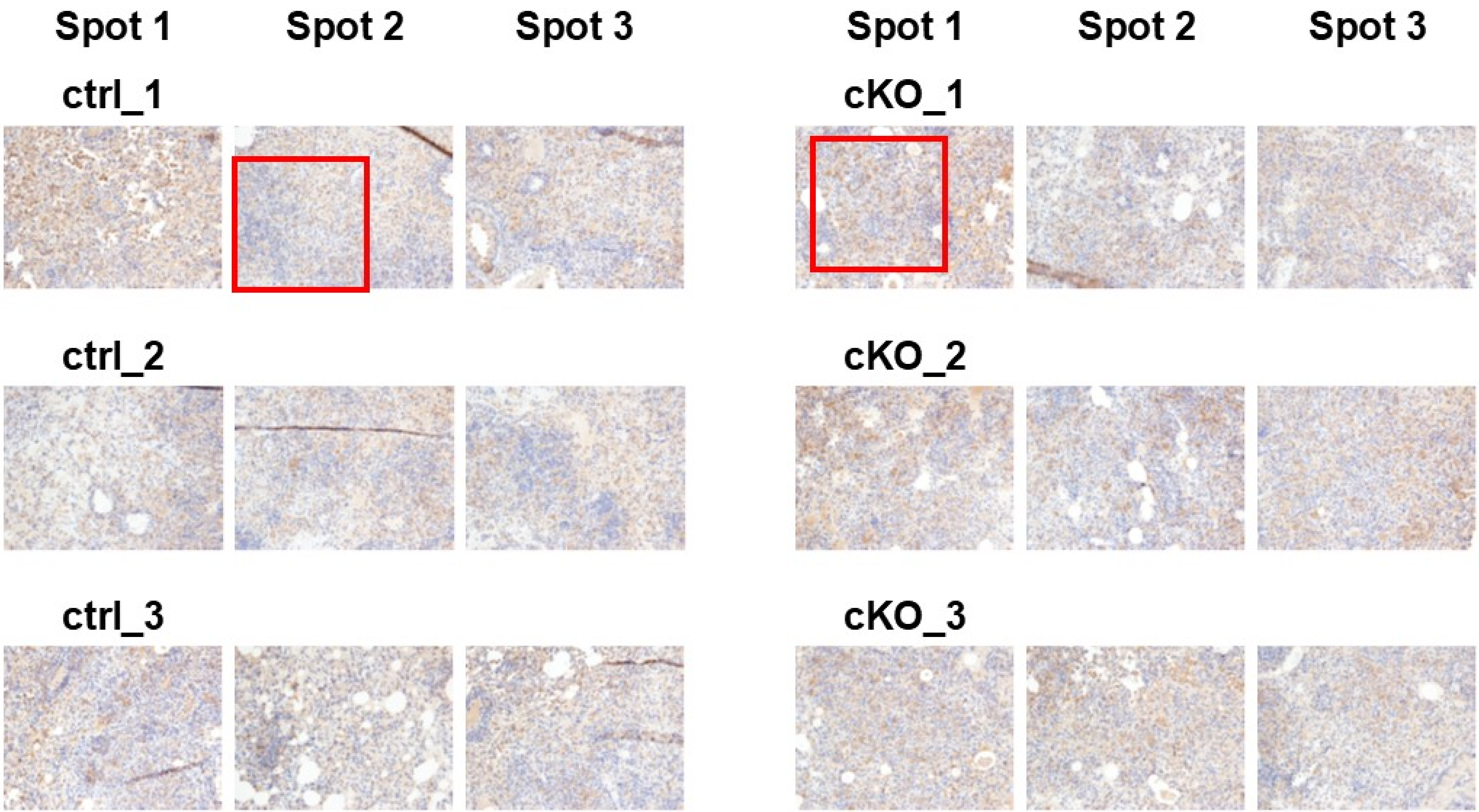
Spot 1
Spot 2
Spot 3
Spot 1
Spot 2
Spot 3
ctrl_1
cKO_1
ctrl_2
cKO_2
ctrl_3
cKO_3
