# Supplementary material for: Mycobacterium tuberculosis impairs protective cytokine production via transcription factor MafB manipulation
Source: PLoS Pathog. 2025 Sep 4;21(9):e1013476. doi: 10.1371/journal.ppat.1013476 (PMC12419584; doi:10.1371/journal.ppat.1013476)
Supplement: Attachment — Submitted filename: Response to Reviewers.docx [file ppat.1013476.s009.docx]

PPATHOGENS-D-25-00053
*Mycobacterium tuberculosis* impairs cytokine production by manipulating transcription factor MafB
PLOS Pathogens

Dear Dr. Saiga,

Thank you for submitting your manuscript to PLOS Pathogens. After careful consideration, we feel that it has merit but does not fully meet PLOS Pathogens's publication criteria as it currently stands. Therefore, we invite you to submit a revised version of the manuscript that addresses the points raised during the review process.

As you can see in the attached reviews, the referees appreciated the importance of the topic and the novelty of your observations.  However, the reviewers agreed that the study was not yet conclusive, and still contained discrepancies that need to be addressed.  Several suggestions were offered to further characterize either the signals regulating MafB, or its downstream effects.  While we do not expect every suggested experiment to be performed, it is clear that significant additional work on these topics is warranted. 

Please submit your revised manuscript within 60 days Apr 08 2025 11:59PM. If you will need more time than this to complete your revisions, please reply to this message or contact the journal office at plospathogens@plos.org. When you're ready to submit your revision, log on to https://www.editorialmanager.com/ppathogens/ and select the 'Submissions Needing Revision' folder to locate your manuscript file.

Please include the following items when submitting your revised manuscript:

* A rebuttal letter that responds to each point raised by the editor and reviewer(s). You should upload this letter as a separate file labeled 'Response to Reviewers'. This file does not need to include responses to any formatting updates and technical items listed in the 'Journal Requirements' section below.
* A marked-up copy of your manuscript that highlights changes made to the original version. You should upload this as a separate file labeled 'Revised Manuscript with Track Changes'.
* An unmarked version of your revised paper without tracked changes. You should upload this as a separate file labeled 'Manuscript'.

If you would like to make changes to your financial disclosure, competing interests statement, or data availability statement, please make these updates within the submission form at the time of resubmission. Guidelines for resubmitting your figure files are available below the reviewer comments at the end of this letter.

We look forward to receiving your revised manuscript.

Kind regards,

Christopher M. Sassetti
Academic Editor
PLOS Pathogens

Michael Wessels
Section Editor
PLOS Pathogens

Sumita Bhaduri-McIntosh
Editor-in-Chief
PLOS Pathogens
orcid.org/0000-0003-2946-9497

Michael Malim
Editor-in-Chief
PLOS Pathogens
orcid.org/0000-0002-7699-2064

**Authors (AU):** We would like to thank the editors for their interest in our paper and for giving us the opportunity to revise the manuscript. All the comments and suggestions provided by the reviewers have significantly contributed to improving the overall quality of our paper. Please find our point-by-point responses, highlighted in blue, below. We also note the general changes here.

**Title changed to:** *Mycobacterium tuberculosis* impairs protective cytokine production via transcription factor MafB manipulation

**Affiliation changed to:** ^2^Research Center for Vaccine Development, National Institute of Infectious Diseases, Japan Institute for Health Security, Tokyo, Japan

^3^Leprosy Research Center, National Institute of Infectious Diseases, Japan Institute for Health Security, Tokyo, Japan

**Acknowledgments:** section updated to include appreciation for Dr. Stefan H. E. Kaufmann (Max Planck Institute for Infection Biology) for his valuable contributions.

**Reviewers' Comments:**

Reviewer's Responses to Questions

**Part I - Summary**
Please use this section to discuss strengths/weaknesses of study, novelty/significance, general execution and scholarship.

Reviewer #1: This paper by Saiga et al works to understand the role of MAFB in M. tuberculosis pathogenesis, as it has been associated with TB susceptibility in genetic studies of Mtb growht. They focus on the role of MAFB in the macrophage in this paper, demonstrating in elegant studies that it inhibits transcription of proinflammatory cytokines by preventing binding of p65 to initiate transcription. Further, they demonstrate that deletion of MAFB selectively in macrophages improves M.tb outcomes and increases the proportion of proinflammatory macrophages in the lung. These observations are interesting and potentially important for understanding m.tb pathogenesis. However, the discussion is sparse and the characterization of MAFB-/- macrophages is incomplete, espeically the role of MAFB in Type I intereforon secretion, as they showed in prior studies that this regulates Type I interferon production in plasmacytoid DC.

Reviewer #2: Saiga et al examine how the transcription factor MafB impacts macrophage cytokine responses and the host response to Mycobacterium tuberculosis. The authors determine that MafB directly interacts with transcription factors IRF5 and PU.1 to inhibit pro-inflammatory cytokines TNF and IL-12p40 and demonstrate, using a conditional knockout mouse model, that the lack of MafB expression in macrophages leads to decreased bacterial burden and increased survival following Mtb infection. The dissection of MafB molecular interactions is particularly strong. The authors generate and use domain mutants for MafB, IRF5, and PU.1 and express these in HEK293T cells to determine direct interactions between transcription factors. They also use both an in vitro model of elicited peritoneal macrophages and an in vivo mouse model using MafB conditional knockout mice to test the role of MafB in macrophages during Mtb infection.

Overall, identifying the factors that regulate whether Mtb-infected macrophages mount a pro-inflammatory response or not is critical to understanding host-pathogen interactions during Mtb infection. Therefore, the overall topic is of significance to the field. This manuscript provides new information about a source of macrophage regulation that was not previously described during Mtb infection. Overall, the studies are well-conceived, with adequate controls. The manuscript is well-written and the experiments are clearly described. However, there are several current limitations to the manuscript that limit enthusiasm, including the rigor of the statistical analysis and some discrepancies between experiments, that need to be addressed.

Reviewer #3: This study investigates the role of the transcription factor MafB in TB pathogenesis, particularly its impact on cytokine production. The authors demonstrate that Mtb upregulates MafB to suppress pro-inflammatory cytokines, such as TNF-α and IL-12p40, thereby facilitating bacterial survival. Using MafB-deficient mice, the study shows enhanced cytokine production, reduced bacterial burden, and improved host resistance, suggesting MafB as a potential biomarker for TB susceptibility. However, the mechanistic basis for MafB regulation remains incompletely defined, particularly regarding the upstream signaling pathways controlling its expression during Mtb infection. Addressing these mechanistic gaps would further solidify the study’s conclusions and translational relevance.

**AU:** We would like to thank all the reviewers for their helpful and constructive comments and feedback on the manuscript. In response to all the reviewers' requests and comments, we have carefully performed several additional experiments, modified the figures, and revised their corresponding citations in the text. We hope you will find our revised manuscript suitable for publication.

**Part II – Major Issues: Key Experiments Required for Acceptance**
Please use this section to detail the key new experiments or modifications of existing experiments that should be absolutely required to validate study conclusions.

Generally, there should be no more than 3 such required experiments or major modifications for a "Major Revision" recommendation. If more than 3 experiments are necessary to validate the study conclusions, then you are encouraged to recommend "Reject".

Reviewer #1: Line Comment
**Request 1**

213 Additional data in macrophages with MAFB overexpression would be good to validate ATRA experiments. It may be that RA induces cytokine responses via an alternate non-MAFB mechanism. Or an experiment that demonstrates that MAFB knockout abolishes ATRA proinflammatory effects
**AU:** As shown in Fig 3F of the revised manuscript, RA stimulation alone does not induce cytokine responses (at least production of TNF-α and IL-12p40). However, as the reviewer pointed out, it is also possible that RA suppresses cytokine responses via an alternate non-MafB mechanisms. Therefore, we performed the same experiment using MafB cKO macrophages. TLR stimulation in the presence of RA hardly suppressed cytokine production in cKO macrophages (**Figure 1 for peer review**), suggesting that RA-induced cytokine reduction is dependent on MafB. Therefore, we concluded that MafB regulates the induction of TNF-α and IL-12p40.

**Figure 1**

**Request 2**

330 What is the breadth of the response in the absence of MAFB? Does MAFB influence other TLR activation? If TLR2 deficient mice do not display worsened m.tb diseaes, why then does MAFb alter responses?

**AU:** Thank you for the valuable comments. We analyzed TLR4 activation in LPS-treated peritoneal macrophages. As shown in the following figure (**Figure 2 for peer review**), MafB could affect the activation of other TLR pathways as well. However, as mentioned later in Request No. 9, we consider that TLR4 signaling has little effect on *M. tuberculosis* infection, because TLR2, but not TLR4, was primarily involved in the induction of pro-inflammatory cytokines in *M. tuberculosis* infection (see Figure 9 for peer review).

Several *in vivo* studies have shown that *Tlr2*-deficient mice are highly susceptible to high-dose *M. tuberculosis* infection, and although this remains controversial, it has been suggested that high susceptibility is attributed to a significant reduction in the production of the pro-inflammatory cytokines TNF-α and IL-12p40 during the early phases of infection (*J Immunol*., 2002, 169:3480–3484., *Am J Pathol.*, 2004, 164:49–57., *Microbes Infect.*, 2006, 8:1790–1800.). We therefore believe that the findings of this paper, suggesting that MafB is a regulatory molecule of pro-inflammatory cytokine production and influences susceptibility to *M. tuberculosis* infection, are consistent with those of previous studies and reasonable.

**Figure 2**

Reviewer #2:

**Request 3**

1) The statistical analysis in the manuscript lacks rigor and critical details. Many bar and line graphs lack error bars. The numbers of technical or biological replicates should be reported for each subfigure. “ * ” statistics shown in all figures do not indicate which comparisons are significant. This information needs to either be provided within the figure via black bars between specific conditions or clearly described within the figure legend or text.

**AU:** Thank you for the valuable comments. We carefully modified the statistical analysis and adapted all data into the figures in the revised manuscript. Additionally, the numbers of technical or biological replicates have been added to all figure legends, including line graphs for which statistical analysis is not available.

**Request 4**

2) The manuscript would benefit from a more robust investigation as to why MafB expression is induced during Mtb infection. The authors wait for the discussion before offering a hypothesis for why MafB expression goes up during Mtb infection, stating that expression of IL-10 might be responsible for induction of MafB. In the discussion, Fig S4 is shared to support this claim. This information should be provided in the main text as part of Fig 4 and it would be helpful to have more data provided to support this claim. For example, does anti-IL-10R treatment block the Mtb-induced MafB expression increase? Why does IL-10-mediated induction of MafB not play a role during TLR stimulation, in which the authors observe that MafB expression decreases, as TLR stimulation can also induce IL-10? The authors provide an alternative hypothesis that nuclear receptors, specifically retinoic acid receptor or PPARg, could induce MafB expression. The authors could similarly test these mechanisms.

**AU:** Thank you for the critical suggestions. In accordance with the reviewer’s requests, we analyzed whether IL-10 is important for MafB expression during *M. tuberculosis* infection using anti-IL-10R (Selleck) to block IL-10 signaling in thioglycolate-elicited peritoneal macrophages. As shown in Fig 4D of the revised manuscript, MafB expression was upregulated by *M. tuberculosis* infection, while anti-IL-10R treatment decreased its expression (**Figure 3 for peer review**). Furthermore, MafB expression was abrogated not only by anti-IL10R but also by nuclear receptor RAR inhibitor (AR7, Selleck) and PPAR-γ inhibitor (GW9662, Selleck) treatment (**Figure 4 for peer review**). In conclusion, we hypothesize that IL-10 and nuclear receptors can be involved in the induction of MafB expression during *M. tuberculosis* infection. Since these results may be informative, we have added these data in Fig 4L-N and S4 Fig of the revised manuscript and re-wrote the Results and Discussion sections accordingly (Lines 290–298, 487–497).

We also compared the IL-10 expression between samples of *M. tuberculosis*-infected and TLR2-stimulated peritoneal macrophages to resolve the discrepancy that TLR-mediated IL-10 does not contribute to increased MafB expression. TLR-induced *Il10* was significantly lower than that during *M. tuberculosis* infection (**Figure 5 for peer review**). These results lead to the conclusion that IL-10 induction could be predominant in *M. tuberculosis* infection, at least in the mycobacteria and TLR2 ligands used in this study.

**Figure 3**

**Figure 4**

**Figure 5**

**Request 5**
3) Line 326: “the MafB expression was not detected in MafB cKO lungs (Fig 5A), indicating that MafB expression in lungs is dependent on tissue macrophages.” The MafB expression data for the cKO lungs was not provided and should be included in Figure 5, if the authors want to make the above claim.

**AU:** Thank you for the helpful comments. We have the older data with newer ones (**Figure 6 for peer review**) and incorporated them into Fig 5A in the revised manuscript. Additionally, we have re-wrote the sentence below to avoid misinterpretation (Lines 361–364).

“MafB mRNA expression was enhanced in the lungs of control mice in the active phase of TB compared with that in uninfected mice, whereas MafB expression was considerably lower in MafB cKO lungs (Fig 5A), indicating that MafB expression in lungs is dependent on tissue macrophages.”

**Figure 6**

**Request 6**
Issues to address in the text:
1) In figure S2A, cKO macrophages have greater Il1b and Il6 expression following TLR2 agonist stimulation but decreased Il1b and Il6 expression following Mtb infection in Fig 4G. The authors should address this discrepancy.

**AU:** Thank you for the comments. As the reviewer pointed out, *Il1b* and *Il6* expression was enhanced in cKO macrophages upon stimulation with TLR2 or TLR4 agonists, but not with *M. tuberculosis* infection (see Fig 4G and S2 Fig in the revised manuscript and Figure 2 for peer review). To address this discrepancy, we additionally performed a luciferase assay with the *Il6* luciferase reporter. We found that, unlike *Tnf* and *Il12b* reporter, IL-6 transcriptional activity was increased by MafB (**Figure 7 for peer review**). For unknown reasons, these findings may be the cause of its discrepancy: the mechanism of transcriptional activity of IL-6 and IL-1β is different from that of TNF-α and IL-12p40. In contrast, other inflammatory genes, Lcn2 and SLPI, were clearly upregulated in cKO macrophages (see S3 Fig in the revised manuscript). Therefore, we re-wrote the relevant parts of the Discussion section to avoid misinterpretation (Lines 445–451).

**Figure 7**

**Request 7**
2) While the molecular dissection of MafB binding in Figures 1 and 2 is very strong, the luciferase reporter assay and immunoprecipitation were performed using HEK293T cells, which may not be the best representative for macrophage intracellular interactions. At minimum, the authors should include a discussion of how the TF interactions might differ between cell types or cell activation states.

**AU:** We agree with the reviewer's concern about the data using HEK293T cells. As noted in Request No. 8 below, although we were able to partially analyze intracellular interactions in peritoneal macrophages and did not observe differences in transcription factor interactions between steady state and the infected state, it was difficult to fully replicate the results with HEK293T cells in a macrophage-based system. We therefore have described the limitations of this experiment in the Discussion section (Lines 457–464).

Reviewer #3: Here are some of my primary concerns, which would require additional experiments.
**Request 8**

1. The study extensively relies on 293T cells to examine MafB’s interactions and transcriptional regulation. While these assays provide mechanistic insights, they lack physiological relevance, as 293T cells do not fully recapitulate macrophage-specific immune responses. Key experiments should be replicated in primary macrophages under Mtb infection conditions to confirm the functional role of MafB in a more relevant context.

**AU:** We understand the reviewer's concern about the data using HEK293T cells. Therefore, we analyzed intracellular interactions in peritoneal macrophages infected or uninfected with BCG; biosafety issues made this experiment difficult to perform under *M. tuberculosis* infection. p65 interacted with IRF5 and MafB bound to both PU.1 and IRF5 within macrophages regardless of BCG infection (**Figure 8 for peer review**). These results are consistent with a previous report (*Cell Rep*., 2014, 8:1308–1317) and with the current data using HEK293T cells. However, the interaction of p65 with MafB and PU.1 with IRF5 could not be accurately analyzed in the macrophage-based system, nor could detailed analysis using mutants be performed. This suggests a limitation of this experiment due to the condition of the antibodies and low transfection efficiency of primary macrophages. Alternatively, the limitations of this experiment have been described in the Discussion section (Lines 457–464).

**Figure 8**

**Request 9**
2. The manuscript does not clarify whether MafB regulation is primarily mediated through TLR2 or TLR4 signaling. Given that TLR4 (activated by LPS) and TLR2 (recognized by Mtb components) initiate distinct immune responses, it is crucial to determine which pathway predominantly controls MafB expression during Mtb infection. Experiments using TLR2- or TLR4-deficient macrophages would be necessary to provide definitive insights.

**AU:** In accordance with the reviewer’s comments, we examined whether TLR2 or TLR4 primarily regulates MafB expression during *M. tuberculosis* infection using each TLR inhibitor (Cu-CPT22; TLR2 inhibitor, Selleck and Resatorvid; TLR4 inhibitor, Selleck) to block TLR signaling in thioglycolate-elicited peritoneal macrophages. mRNA expression of pro-inflammatory cytokines was enhanced in *M. tuberculosis* infection when TLR4 signaling was blocked, but not when TLR2 signaling was blocked, suggesting that TLR2 is mainly responsible for cytokine induction in *M. tuberculosis* infection (**Figure 9 left, for peer review**). Under these conditions, we investigated the expression of MafB and found that its expression was equally upregulated by *M. tuberculosis* infection in the presence of each inhibitor (**Figure 9 right, for peer review**). These results indicate that TLRs are not involved in at least the upregulation of MafB expression by *M. tuberculosis* infection.

**Figure 9**

**Request 10**
3. The study reports that TLR2 stimulation with Mtb-derived components decreases MafB levels while increasing TNF-α and IL-12p40 production. However, during live Mtb infection, MafB expression instead increases. Since Mtb contains TLR2 agonists, this apparent contradiction needs further clarification. Does Mtb actively counteract TLR2-induced MafB suppression through additional virulence mechanisms? Investigating whether Mtb employs alternative pathways to upregulate MafB would help reconcile these findings.

**AU:** As mentioned in the Discussion section, several TLR stimuli and low-dose *M. tuberculosis* infections (probably due to the TLR2 agonists harbored by *M. tuberculosis*) suppressed MafB expression (see Fig 4A and D in the revised manuscript). In contrast, *M. tuberculosis*-inducible IL-10 and the nuclear receptors RAR and PPAR-γ, which have been reported to be involved in the induction of MafB expression, appear to contribute to the enhancement of MafB expression in *M. tuberculosis* infection (see Fig 4L-N and S4 Fig in the revised manuscript and Figures 3-5 for peer review). Among these, we believe that *M. tuberculosis*-inducible IL-10 is a strong candidate for direct triggering of MafB expression. Interestingly, a recent study demonstrated that inhibition of triggering receptor expressed on myeloid cells 2 (Trem2), which mediates anti-inflammatory immune signaling, reduces microglial populations with high MafB expression (*Proc Natl Acad Sci U S A*, 2025, 122:e2426786122.). Other studies have reported that Trem2 contributes to *M. tuberculosis* survival by promoting IL-10 production, suppressing pro-inflammatory cytokine secretion, and limiting macrophage activation against mycobacteria in a Trem2-dependent manner (*mBio*, 2022, 13:e0145622., *Nat Commun*., 2021, 12:2299.). Consistent with these findings and our data, we propose that Trem2-mediated signaling, including IL-10 induction, may be responsible for upregulating MafB expression during *M. tuberculosis* infection. Since these suggestions may be informative, we have incorporated them into the Discussion section (Lines 487–497).

**Request 11**
4. The manuscript states that TNF-α is undetectable in in vivo experiments, yet neutralizing TNF-α has a significant impact on bacterial burden. This raises concerns about the detection method used—was the sensitivity of the assay sufficient? Alternatively, could other factors, such as localized TNF-α production within granulomas, explain this discrepancy?

**AU:** Thank you for your critical comments. We believe that there is no problem regarding the sensitivity of the assay because the detection of TNF-α concentration in the blood of infected mice was performed using an ELISA kit (BioLegends) often used in *in vitro* experiments, and a significant amount of the serum was used for the analysis. Furthermore, measurements with another ELISA kit (BD Biosciences) showed similar results, leading us to conclude that TNF-α in the blood is difficult to detect. We therefore additionally examined the expression of *Tnfa* and *Il12p40* in the lungs of mice 4 weeks post-infection. mRNA expression of TNF-α and IL-12p40 was significantly elevated in MafB cKO lungs compared to expression in control mice (**Figure 10 for peer review**), supporting that mycobacteria limit cytokine production by enhancing MafB expression. Since these results may be informative, we have added these data to Fig 5B of the revised manuscript.

**Figure 10**

**Request 12**
5. The study does not address whether TNF-α or IL-12p40 plays a more dominant role in MafB-mediated immune suppression. Since both cytokines are critical for TB immunity, it would be valuable to assess the effects of individual vs. combined neutralization of TNF-α and IL-12p40 in MafB-deficient mice. This could help determine whether one cytokine is the primary driver of resistance in the absence of MafB. Additionally, does TNF-α or IL-12p40 neutralization in wild-type mice fail to enhance Mtb pathogenicity?

**AU:** In accordance with the reviewer’s comments, we examined the effect of combined neutralization of TNF-α and IL-12p40 in *M. tuberculosis*-infected mice. As shown in the following figure (**Figure 11 for peer review**), TNF-α alone or combined neutralization had the same effect on mice, while the single neutralization of IL-12p40 had a milder effect. This may suggest a greater contribution of TNF-α to host defense in acute mycobacterial infection. Furthermore, the impact of neutralizing antibodies on the virulence of *M. tuberculosis* is unknown, but the pathogenesis seems to be enhanced in antibody-treated mice compared to isotype control mice.

**Figure 11**

**Part III – Minor Issues: Editorial and Data Presentation Modifications**
Please use this section for editorial suggestions as well as relatively minor modifications of existing data that would enhance clarity.

Reviewer #1:

**Request 13**

The discussion is cursory and does not provide context for several issues of interest -- why does MAFB improve MTB outcomes when other adaptors that increase immune responses, like TOLLIP (Venkatasubramanian et al Nat Micro 2024) or variants in IL1B (Zhang et al Plos Pathogens 2014), do not? Does the mouse model recapitulate findings in human populations -- have MAFB been shown to have a functional variant and does the directionality of effect between mice and humans correlate? If not, why not?

**AU:** Thank you for the complex but significant suggestions. Several studies highlight type I IFNs as a key factor in TB exacerbation and, although controversial, the involvement of IL-1β in type I IFNs induction has also been reported (*eLife,* 2021, 10:e67290., *Nature*, 2010, 466:973–977., *Front. Immunol*., 2017, 8:1633., *Molecular Cell*, 2019, 74:801–815.). In our current study, *M. tuberculosis*-infected MafB cKO macrophages have not shown an elevated expression of IL-1β and type I IFNs compared with WT macrophages (see Fig 4G in the revised manuscript and Figure 12 for peer review). We therefore hypothesized that the difference of IL-1β production might contribute to this discrepancy for TB susceptibility between these findings and MafB cKO. Additionally, the pleiotropic effect of MafB, such as its influence on the expression of a wide range of inflammatory effectors including not only TNF-α and IL-12p40 but also the mycobactericidal inflammatory genes Lipocalin 2 and SLPI (*J Immunol*., 2008, 181:8521–8527., *J Immunol*., 2008, 180:4032-4039., *Clin Dev Immunol*., 2011, 2011:347594.), may also affect TB susceptibility. Various rare diseases, such as multicentric carpotarsal osteolysis syndrome (MCTO) and focal segmental glomerulosclerosis (FSGS), are known to be caused by point mutations in the MafB gene. Mouse models with point mutations (p.Leu239Pro, p.Pro59Leu) in the MafB gene have been reported to develop similar symptoms and simulate several features of human diseases with relatively high fidelity (*Biochem Biophys Res Commun*., 2020, 523:452–457., *Exp. Anim*., 2019, 68:103–111.). Current mouse studies have also replicated an elevated MafB expression in patients with TB, suggesting a correlation between mice and humans. Thus, further elucidation of the pathology using MafB mouse models could contribute to the generation of novel treatments for these diseases as well as for TB. We have added the corresponding information in the Discussion section (Lines 522–526).

**Request 14**
Recently, observation were made that excess Type I interferon was detrimental for Mtb control (Ji et al Nat Micro 2019). Given that MAFB deficiency in macropahges is beneficial with increased TNF
Does MAFB induce increased Type I inteferon in macrophages after Mtb infection as it does with pDC? This may be key to understanding why increased cytokines in this model are beneficial while they are detrimental in other models

**AU:** In our previous paper, we showed that MafB negatively regulates type I IFN production in pDCs (*Int Immunol*., 2022, 34:159–172.). To investigate whether MafB is also involved in the regulation of type I IFN production in macrophages, we analyzed the expression of type I IFNs using TLR2-stimulated or *M. tuberculosis*-infected macrophage samples. As shown in the following figure (**Figure 12 for peer review**), neither *Ifna* nor *Ifnb* expression was consistent between TLR2 and *M. tuberculosis* and differed from the feature of *Tnfa* and *Il12p40* expression. This may mean that the transcription factors involved in type I IFN induction in macrophages are different from those of TNF-α and IL-12p40 and from the regulatory mechanisms in pDCs. Therefore, we concluded that type I IFNs are not involved in the beneficial effect of *Mafb* deficiency on *M. tuberculosis* control.

**Figure 12**

Reviewer #2:

**Request 15**

1) Line 92: change “molecular basis evidence” to “molecular-based evidence”
**AU:** Thank you for pointing this out. We have deleted this phrase accordingly.

**Request 16**
2) The macrophages used in Figures 3 and 4 should be correctly described as “thioglycolate-elicited peritoneal macrophages” or “elicited peritoneal macrophages”.
**AU:** Thank you for the pertinent comments. We have revised “peritoneal macrophages” to “thioglycolate-elicited peritoneal macrophages” in Figs 3 and 4 accordingly.

**Request 17**
3) The different conditions in 5G are difficult to see, especially the “cKO + anti-TNFa” condition, which is hidden behind the other conditions.
**AU:** We agree with your comment. For ease of viewing, we divided the graph into isotype control groups, anti-TNFα antibody-treated groups, and anti-IL12p40 antibody-treated groups (**Figure 13 for peer review**). We have added a new version in Fig 5H of the revised manuscript.

**Figure 13**

**Request 18**
4) Pathology and lesions in 5E would benefit from quantification, if possible.
**AU:** Thank you for the meaningful suggestions. Although it was very difficult to quantify Iba1 and Ziehl-Neelsen staining panels, our pathologist colleagues helped quantify them. We have included these informative quantification data in S5 Fig of the revised manuscript due to the limited space in the text.

**Request 19**
5) Figure 6 should depict the role of MafB during Mtb infection (in addition to TLR stimulation).

**AU:** Thank you for the helpful suggestions. In accordance with the reviewer’s request, we have included a schematic diagram illustrating events during *M. tuberculosis* infection (**Figure 14 for peer review**) and incorporated it into Fig 6 in the revised manuscript.

**Figure 14**

Reviewer #3: none.

PLOS authors have the option to publish the peer review history of their article ([what does this mean?](https://track.editorialmanager.com/CL0/https:%2F%2Fjournals.plos.org%2Fplospathogens%2Fs%2Feditorial-and-peer-review-process%23loc-peer-review-history/1/010f0194dfde772e-f6bd2396-1e0c-4690-913c-8da9ddbc2bf0-000000/tWoQDqtbl2WYIzDRMU0AZQQTNwLPgvMq_1xwoTd7nxc=197)). If published, this will include your full peer review and any attached files.


If you choose “no”, your identity will remain anonymous but your review may still be made public.


**Do you want your identity to be public for this peer review?** For information about this choice, including consent withdrawal, please see our [Privacy Policy](https://track.editorialmanager.com/CL0/https:%2F%2Fwww.plos.org%2Fprivacy-policy/1/010f0194dfde772e-f6bd2396-1e0c-4690-913c-8da9ddbc2bf0-000000/PeDR4aIDSlDsvBJKppZoJFZXVNY6fO6HVMeex2pPMxo=197).

Reviewer #1: No

Reviewer #2: No

Reviewer #3: No

[NOTE: If reviewer comments were submitted as an attachment file, they will be attached to this email and accessible via the submission site. Please log into your account, locate the manuscript record, and check for the action link "View Attachments". If this link does not appear, there are no attachment files.]

**Figure resubmission:**

While revising your submission, please upload your figure files to the Preflight Analysis and Conversion Engine (PACE) digital diagnostic tool, https://pacev2.apexcovantage.com/. PACE helps ensure that figures meet PLOS requirements. To use PACE, you must first register as a user. Registration is free. Then, login and navigate to the UPLOAD tab, where you will find detailed instructions on how to use the tool. If you encounter any issues or have any questions when using PACE, please email PLOS at figures@plos.org. Please note that Supporting Information files do not need this step. If there are other versions of figure files still present in your submission file inventory at resubmission, please replace them with the PACE-processed versions.

**Reproducibility:**

To enhance the reproducibility of your results, we recommend that authors of applicable studies deposit laboratory protocols in protocols.io, where a protocol can be assigned its own identifier (DOI) such that it can be cited independently in the future. Additionally, PLOS ONE offers an option to publish peer-reviewed clinical study protocols. Read more information on sharing protocols at https://plos.org/protocols?utm_medium=editorial-email&utm_source=authorletters&utm_campaign=protocols

**AU:** We would like to express our sincere gratitude to the editors and reviewers for their positive and constructive criticism. The manuscript has vastly benefited from your valuable and insightful comments and suggestions. We look forward to hearing from you and would be happy to address any further concerns, if required. We hope this further pushes the manuscript closer to publication in your esteemed journal.
